# Visible-Near-Infrared Hyperspectral Imaging Enables Nondestructive Identification of Bean Accessions via 1D Spectral Reflectance Analysis

**DOI:** 10.1021/acsomega.6c00225

**Published:** 2026-05-07

**Authors:** Renan Falcioni, Nicole Ghinzelli Vedana, Caio Almeida de Oliveira, João Vitor Ferreira Gonçalves, José Alexandre M. Demattê, Marcos Rafael Nanni

**Affiliations:** † Graduate Program in Agronomy, 42487 State University of Maringá, Av. Colombo, 5790, Maringá 87020−900, Paraná, Brazil; ‡ Department of Biology, 42487 State University of Maringá, Av. Colombo, 5790, Maringá 87020−900, Paraná, Brazil; § Department of Soil Science, Luiz de Queiroz College of Agriculture, 54538 University of São Paulo, Av. Pádua Dias, 11, Piracicaba 13418−260, São Paulo, Brazil

## Abstract

Reliable seed accession identification via sensors underpins germplasm conservation, traceability, and breeding, yet conventional assays are often destructive, labor-intensive, and difficult to scale. Here, visible-near-infrared (VNIR) hyperspectral imaging (Headwall Photonics, 825 bands) was used to obtain one ROI-averaged 1D reflectance spectrum per seed and to classify 32 grain-legume accessions (*N* = 3200 seeds; 100 seeds per accession), comprising 30 common bean (*Phaseolus vulgaris* L.) landraces and two locally co-classified outgroup legumes (*Vigna angularis* (Willd.) Ohwi and H. Ohashi and *Cajanus cajan* (L.) Huth). The seed-level reflectance signatures exhibited a strong accession structure (PERMANOVA; *F* = 201.40, *p* < 0.001). Principal component analysis (PCA) captured 94.48% of the total spectral variance in the first three components (PC1 = 67.17%, PC2 = 21.80%, PC3 = 5.51%), indicating that discrimination is dominated by smooth, wavelength-contiguous modes consistent with color-related absorption and scattering-mediated optics. Wavelength-resolved one-versus-rest association identified accession-specific informatdive bands, with the strongest single association reaching *r* ^2^ = 0.736 at 424.79 nm, whereas supervised ReliefF ranking concentrated multiclass informativeness in a narrow green window (562.85–584.65 nm; peak ≈ 581.74 nm), providing direct guidance for reduced-band multispectral screening; however, reduced-band tests indicate that this window alone is insufficient for the full 32-class task. In full-spectrum multiclass modeling, classical learners performed best, with a linear support vector machine (Linear SVM) achieving 88.59% accuracy on the independent 20% test split (weighted F1-score = 88.52%) and exhibiting structured confusions among spectrally adjacent accessions. One-dimensional deep learning models were evaluated only as a secondary benchmark; the best network (MLP_1D) reached 82.03% test accuracy (weighted F1-score = 81.52%) but did not exceed the strongest classical model under this mean spectrum, moderate-data regime. The green window is interpreted as an optical leverage region linked to seed coat color and pattern and surface microstructure, although no targeted chemical assays were performed in this study. Because only one seed lot per accession was analyzed under controlled laboratory conditions, robustness to storage conditions, seed moisture content, and harvest time remains to be tested. Overall, VNIR hyperspectral imaging coupled with interpretable 1D spectral reflectance analysis provides a practical, mechanistically plausible route to accession-level seed identification and can inform reduced-band sensor designs for scalable screening in food and agriculture.

## Graphical abstract

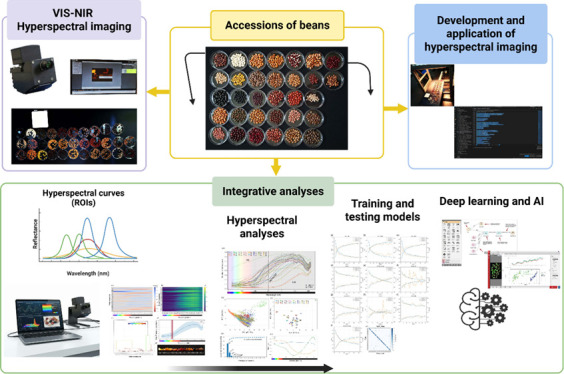

## 1 Introduction

Common bean (*Phaseolus vulgaris* L.) and other grain legumes remain central to food security and culinary identity in Brazil and worldwide, yet their value chains still depend heavily on visual inspection and informal naming conventions that do not scale to large, mixed, or processed lots.^1,2^ This creates a persistent vulnerability to mislabeling, loss of landrace identity, and inconsistent product quality, particularly when morphologically similar seeds co-occur or when minor phenotypic differences carry major cultural and economic meaning.^1–4^ Recent work on bean and other legume seed quality and authenticity highlights both the demand for rapid screening and the limits of purely macroscopic descriptors when chemical composition, processing behavior, and provenance are the true targets of quality control in agricultural practices.^5–8^

Hyperspectral imaging in the visible–near-infrared (VNIR) range offers a nondestructive route to quantify seed phenotypes at scale while retaining a direct link to optical physics.^9^ In agricultural systems, hyperspectral pipelines have matured from exploratory phenotyping to operational prediction tasks, with a growing emphasis on reproducibility, robustness, and deployment constraints.^10^ For seeds in particular, the promise is mechanistic: reflectance is shaped by visible absorption (color-related) in the testa, by wavelength-dependent scattering driven by the surface microstructure and internal interfaces, and by near-infrared signatures linked to O–H and C–H chemistry and effective path length.^2,11,12^ Seed-focused reviews increasingly argue that hyperspectral measurements are most valuable when spectral differences can be tied to interpretable traits rather than treated as anonymous high-dimensional fingerprints.^7,13–15^

Machine learning has become the default interface between hyperspectral signals and actionable decisions, but most current practice remains correlational.^8,11,16,17^ For ROI-averaged seed spectra such as those used here, the central question is whether the discriminative signal can already be captured by low-dimensional descriptive analysis and comparatively simple classifiers, particularly the PCA-supported structure and linear decision boundaries, rather than by high-capacity architectures. One-dimensional deep learning networks can still be compared as a secondary benchmark, but they do not define the primary scope of the present study.

This is a mechanistic gap with practical consequences.^8,11,24^ If the discriminative signal is dominated by visible absorption–scattering regimes associated with the seed coat color and surface microstructure, then model performance should be explainable through a smooth, wavelength-contiguous structure and should be concentrated in optically meaningful regions of the spectrum.^11,25^ If, instead, discrimination relies on idiosyncratic correlations, then accuracy will degrade under modest shifts in illumination, sensor drift, seed orientation, or storage state, even when cross-validation appears strong.^8,24,26^ Recent work on explainable artificial intelligence for spectroscopy further argues that explanations should function as physical consistency checks^27–29^ rather than post hoc decorations and therefore must be used to test whether learned decision cues remain compatible with plausible optical mechanisms.^13,14,21–23,30^ Despite the rapid adoption of hyperspectral imaging across food and seed applications, reliable deployment remains challenging because discriminative spectral signals can be subtle relative to within-lot variability and are sensitive to illumination, calibration drift, and sample presentation. Recent reviews emphasize that robust data modeling, validation, and interpretation are critical bottlenecks in hyperspectral pipelines for ensuring seed and food quality and safety.^19,20^

A second, related gap concerns sensor design and throughput.^31^ Full hyperspectral systems provide dense spectral sampling, but operational screening often requires reduced-band multispectral solutions that are less expensive, faster, and easier to maintain.^32–34^ Band-selection research has progressed quickly, yet many approaches still optimize separability without articulating why particular windows should remain stable biomarkers across conditions.^35^ Without a mechanistic rationale, wavelength reduction risks discarding precisely the information needed to distinguish visually similar landraces or to detect and separate outgroup legumes that are locally traded under the same category.^18,30,36–38^

Finally, moving from correlation to mechanism increasingly requires causal and probabilistic thinking, not merely larger models.^10,39^ Contemporary surveys of causal machine learning emphasize that robust generalization depends on modeling invariances and explicitly accounting for confounding factors.^15,40,41^ This perspective also highlights interventions and uncertainty, especially when observational data contain acquisition- or storage-related factors that can masquerade as biological signals.^42^ In digital agriculture, recent works argue that causality and explainability should be treated as a single design constraint for trustworthy decision support. This is because stakeholders must know both what drives a prediction and how it might change under plausible actions or shifting environments.^10,39^ For hyperspectral seed authentication, this implies that the central problem is not only accurate classification but also the identification of stable, physically grounded spectral mechanisms that remain informative when nuisance variation is introduced.^5–7,43^

In this study, we develop a controlled VNIR hyperspectral benchmark comprising 30 traditional Brazilian common bean (*Phaseolus vulgaris* L.) landraces and two non-Phaseolus grain legumes locally co-classified as “Feijões.” We analyze full-spectrum reflectance via an interpretable workflow restricted to one central ROI-averaged 1D spectrum per seed, integrating low-dimensional variance structure, wavelength-wise association patterns, and supervised feature ranking. Classical models, especially PCA-supported structure and linear SVM classification, form the primary analytical focus, whereas one-dimensional deep networks are included only as a secondary comparative benchmark. The primary objective is to test whether accession identity can be predicted from VNIR reflectance spectra at the seed level and whether informative wavelength regions can be identified to guide reduced-band screening. We hypothesize that discrimination is dominated by visible-range seed coat absorption/scattering effects consistent with seed coat color differences and by scattering-related albedo shifts, yielding structured, reproducible error patterns concentrated among optically similar accessions rather than stochastic misclassification.

## 2 Material and Methods

### 2.1 Plant Material, Genetic Background, and Experimental Design

Thirty-two grain-legume accessions were included in the study. Thirty accessions corresponded to traditional common bean (*Phaseolus vulgaris* L.) landraces, and two accessions were non-*Phaseolus* grain legumes that are locally handled and classified as “Feijões” within Brazilian food systems, namely, A27: *Vigna angularis* (Willd.) Ohwi & H. Ohashi and A28: *Cajanus cajan* (L.) Huth (Table S1).

The selected landraces represent a broad spectrum of traditional bean types in Brazil, encompassing substantial phenotypic, biochemical, and genetic diversity preserved through long-term farmer selection and regional adaptation processes. The collection included the following accessions: (1) Feijão Amendoim Roxo, (2) Feijão Carioca, (3) Feijão Preto Gigante, (4) Feijão Branco Manteiga, (5) Feijão Rosinha, (6) Feijão Branco Gigante, (7) Feijão Canário, (8) Feijão Preto Mocotó, (9) Feijão Verde, (10) Feijão Vermelho Bolinha, (11) Feijão Branco Bolinha, (12) Feijão Mouro Gigante, (13) Feijão Roxinho, (14) Feijão Creme, (15) Feijão Vermelho Gigante, (16) Feijão Jalo, (17) Feijão Amendoim Bege, (18) Feijão Boreal Rosa, (19) Feijão Chocolate, (20) Feijão Amendoim Rosa, (21) Feijão Olho de Pombo, (22) Feijão Bico de Ouro, (23) Feijão Amendoim, (24) Feijão Ovo de Tito-Tico, (25) Feijão Amendoim Verde, (26) Feijão Rajado, (27) Feijão Azaki, (28) Feijão Guandu, (29) Feijão Mouro, (30) Feijão Mancá, (31) Feijão Vermelho and (32) Feijão Rapa Cuiua (Table S1).

For each accession, 100 intact seeds were used to standardize a balanced design (*N* = 3200). When larger lots were available, 100 seeds were randomly selected from the lot; seeds with visible damage were excluded to avoid artifactual spectra.

Because seeds are nested within accessions and each accession originates from a single distinct seed lot, inferential statements are interpreted as describing the separability within the sampled lots rather than across multiple harvests or storage histories. Storage conditions, seed moisture content, and harvest time were not experimentally varied in this benchmark. We therefore report both seed-level metrics and an accession-level (majority-vote) identification accuracy computed from multiple seeds per accession. Accordingly, uncertainty in the performance metrics was quantified via accession-clustered resampling (e.g., cluster bootstrap by accession), and the results were interpreted at both the seed level (*N* = 3200) and the accession level (*G* = 32).

### 2.2 Hyperspectral Image Acquisition and Spectral Consistency Analysis

Hyperspectral imaging was performed via a Headwall Photonics A-Series (380–1100 nm) push-broom hyperspectral system (Headwall Photonics Inc., Bolton, MA, USA) mounted on a laboratory scanner platform configured for controlled push-broom acquisition. The calibrated wavelength axis of the range retained for analysis spanned the 423.34–1,022.07 nm range, yielding *P* = 825 contiguous spectral bands with a uniform sampling interval (∼0.73 nm; spectral resolution).

Data were acquired over four scanning sessions, with each session comprising multiple Petri dishes and contributing to the full set of *N* = 3200 seeds. For each scan, approximately 50 seeds were arranged in a single layer in Petri dishes on a stable, low-reflectance background to minimize overlap and reduce background mixing during ROI definition. The sensor-to-sample distance was fixed at 85 cm to standardize spatial sampling and reduce geometry-driven variability. Acquisition was performed under controlled laboratory conditions; twelve 20 W halogen lamps were evenly placed around the scan window to provide uniform illumination, the ambient temperature was maintained at 25 °C, and external light interference was eliminated. Camera cooling was enabled, and the system was allowed to thermally stabilize prior to acquisition to reduce drift and electronic noise. Reflectance calibration relied on a diffuse Spectralon (Labsphere Inc., North Sutton, NH, USA) reference target and dark-current measurements, enabling conversion from raw intensity to relative reflectance values.

#### 2.2.1 Reflectance Conversion

Before each acquisition session, dark-current reference frames were collected with illumination disabled to characterize the sensor noise and fixed-pattern components. White reference frames were acquired via a certified Spectralon diffuse reflectance panel (99% reflectance; Labsphere Inc., North Sutton, NH, USA). The acquisition parameters were defined in the manufacturer’s software (Hyperspace III, Headwall Photonics), which uses an integration time of 39 ms, a frame period of 40 ms, and a translation speed of 50 mm min^–1^, providing continuous line–scan coverage without spatial discontinuities.

Pixelwise reflectance was computed by subtracting the dark reference and normalizing it to the dark-corrected white reference, producing physically interpretable relative reflectance values for subsequent spectral analysis.

#### 2.2.2 Region of Interest Extraction and Spectral Signature Generation

After reflectance calibration, the hyperspectral cubes were exported in ENVI Classic software, version 5.4 (Harris Geospatial Solutions, Boulder, CO, USA) for downstream processing. For each seed, a single region of interest (ROI) was delineated to capture a consistent central area while avoiding edge pixels with curvature and mixed background contributions or specular artifacts that could bias the reflectance. Each ROI comprised a 10 × 10-pixel square (100 pixels), and the mean reflectance at each wavelength was computed across ROI pixels to produce one 1D spectrum per seed. This procedure generated seed-level spectral signatures associated with accession labels (100 spectra per accession). The 10 × 10-pixel ROI size was selected as a compromise between (i) capturing enough within-seed area to average down sensor noise and local texture and (ii) avoiding edge curvature, specular highlights, and background mixing. This choice was confirmed by pilot visual inspection across accessions to ensure that the ROI remained fully inside the seed body for all morphologies. The inspection also verified that the reflectance curves were stable under small within-seed shifts of ROI placement in a pilot subset (three candidate central placements), supporting the use of a single ROI per seed in the final data set. No pixelwise spatial descriptors, seed-shape measurements, or color-heterogeneity features were extracted; the study is intentionally restricted to one central ROI-averaged 1D reflectance spectrum per seed.

### 2.3 Organization of the Spectral Data Set and Python Implementation

All analyses following spectral extraction were implemented in Python version 3.13.9 scripts within a single script workflow to ensure reproducibility. The reflectance data were organized as a matrix $X\in R^{N\times P}$ , where *N* = 3200 denotes the number of ROI-level spectra and where *P* = 825 denotes the number of wavelength bands. Each row *x*_*i*_ corresponds to the mean reflectance spectrum of ROI *i*, and each spectrum is associated with an accession label *y* ∈ {*A*01, ···, and *A*32}^*N*^. The wavelength axis was stored as λ = (λ_1_, ···, and λ_*P*_)^T^, with λ_*j*_ expressed in nm. Conventional machine learning procedures were implemented via standard Python ML tooling.^44^

### 2.4 Spectral Preprocessing

These preprocessing steps are common in VNIR hyperspectral seed analysis pipelines.^33^ Accessions were indexed via codes A01–A32, corresponding directly to the cultivar numbering defined in Section 2.1 and reported in Table S1.

Polynomial baseline correction (degree 2) was available but not applied in the reported analyses, as reflectance spectra were obtained via white-reference calibration. The spectra were smoothed via a Savitzky–Golay filter (window length = 11, polynomial order = 2) to reduce high-frequency noise while preserving diagnostically relevant band-shaped features.

Standard normal variate (SNV) normalization was implemented as an optional procedure but was not employed in the primary analyses, as it rescales spectra into a centered and variance-normalized space that can include negative values and therefore is not directly interpretable as reflectance. When applied for sensitivity analysis, SNV was defined as

$$x_{\mathrm{ij}}^{\prime }=\frac{x_{\mathrm{ij}}-\mu_{i}}{\sigma_{i}}$$

where μ*i* and σ*i* denote the mean and standard deviation of spectrum *i*, respectively. After smoothing, the reflectance values were constrained to the interval [0, 1] by clipping to ensure physical plausibility.

### 2.5 Descriptive and Integrative Spectral Analyses

Exploratory analysis was used to characterize between-accession spectral variability and to identify wavelength regions associated with accession differentiation. Accession-wise mean spectra were computed across the full wavelength range and visualized to evaluate systematic differences in visible reflectance. We also examined the near-infrared region, where reflectance is strongly influenced by scattering regimes and broad absorptive behavior. Global spectral (VNIR) differences among accessions were tested via permutational multivariate analysis of variance (PERMANOVA) applied to the Euclidean distance matrix of preprocessed spectra, with accession as the grouping factor and 999 permutations, yielding an F statistic and a permutation *p*-value.

To quantify wavelength-specific associations with accession identity, Pearson correlation coefficients were computed between the reflectance values at each wavelength and the one-versus-one-resistance encoded accession labels. Here, labels were encoded as binary indicators (1 = target accession; 0 = all other accessions), which treats accession identity as a nominal variable and does not impose any ordering or numeric distance between accession IDs.

The Pearson correlation coefficient was defined as

$$r=\frac{\overset{N}{\underset{i=1}{\sum }}\left(x_{i}-\mathrm{x̀}\right)\left(z_{i}-\mathrm{z̀}\right)}{\sqrt{\overset{N}{\underset{i=1}{\sum }}{\left(x_{i}-\mathrm{x̀}\right)}^{2}}\sqrt{\overset{N}{\underset{i=1}{\sum }}{\left(z_{i}-\mathrm{z̀}\right)}^{2}}}$$

where *x*_*i*_(λ) is the reflectance of seed i at wavelength λ, *x*(λ̅) is the mean reflectance at λ across all seeds, *z*_*i*, *c*_ ∈ 0,1 indicates whether seed i belongs to accession *c* (one-versus-rest coding), and *z*_*c*_ is the mean of *z*_*i*, *c*_ across seeds (*i* = 1, ···, *N*).

Hierarchical clustering was applied to the accession-averaged spectra via Euclidean distance:

$$d\left(a,b\right)=\sqrt{\overset{P}{\underset{j=1}{\sum }}{\left(x_{a,j}-x_{b,j}\right)}^{2}}$$

where *x*_*a*, *j*_ denotes the mean reflectance of accession *a* at wavelength index *j*.

### 2.6 Principal Component Analysis

Principal component analysis (PCA) was used to obtain a compact representation of the dominant modes of hyperspectral variability and to support the mechanistic interpretation of the variance structure. Prior to PCA, the spectra were standardized bandwise to zero mean and unit variance:

$$X_{s,ij}=\frac{X_{ij}-\mu_{j}}{\sigma_{j}}$$

where

$$\mu_{j}=\frac{1}{N}\overset{N}{\underset{i=1}{\sum }}X_{ij},\sigma_{j}=\sqrt{\frac{1}{N-1}\overset{N}{\underset{i=1}{\sum }}{\left(X_{ij}-\mu_{j}\right)}^{2}}$$

PCA was applied to the standardized matrix to obtain score and loading representations.

The explained variance ratios were exported for the first 20 components, and both individual-spectrum and accession-centroid score plots were generated. Loading vectors (PC1–3) were analyzed to identify the wavelength regions contributing most strongly to principal component separation.

### 2.7 Feature Ranking and Selection via ReliefF

Feature ranking was used to quantify which wavelengths contributed most to accession separability and to highlight compact spectral regions that could inform future reduced-band multispectral screening sensors:

$$w_{j}\leftarrow w_{j}-\frac{1}{mk}\underset{h\in H_{i}}{\sum }\left|x_{ij}-x_{hj}\right|+\frac{1}{mk}\underset{m\in M_{i}}{\sum }\left|x_{ij}-x_{mj}\right|$$

where *w*_*j*_ is the ReliefF weight for wavelength band *j*, *m* is the number of sampled reference spectra (iterations), *k* is the number of nearest neighbors, *H*_*i*_ *s* the set of *k* nearest hits (same accession as spectrum (*i*), *M*_*i*_ *s* the set of *k* nearest misses (different accessions), and *x*_*sj*_ denotes the reflectance of spectrum *s* at band *j* (*s* = *i*, *h*, *r*).

To identify the wavelength regions that contribute most to accession separability and to provide deployability-relevant guidance for reduced-band sensor design, a ReliefF feature-ranking procedure was applied to the preprocessed spectra. In each iteration, the algorithm compares a randomly sampled spectrum to its nearest neighbors within the same class (hits) and to nearest neighbors from other classes (misses) and updates a weight for each wavelength by penalizing within-class differences and rewarding between-class separation. The resulting weight vector $w\in R^{P}$ was used to rank wavelengths and visualize the most informative bands on the mean spectrum, linking discrimination to interpretable spectral regions. Wavelength ranking is reported for interpretation and sensor-design insight; it was not used to restrict the feature space during the primary model benchmarking, and all predictive models reported in the main benchmark were trained on the full 825-band spectra.

To verify reduced-band deployability, we performed a sensitivity analysis in which the top-performing classical model (linear SVM with bandwise standardization) was retrained using (i) only the top *K* = 16 wavelengths ranked by ReliefF, (ii) only the green interval 562.85–584.65 nm, and (iii) uniformly downsampled spectra spanning the full VNIR range (sampling steps of 5–30 nm). All reduced-band models were evaluated under the same fixed stratified 80/20 hold-out split used in the main benchmark. The reduced-band results are reported in Table S4.

#### 2.7.1 ReliefF Implementation Details and Hyperparameters

ReliefF feature weighting was implemented in Python using NumPy for weight updates and scikit-learn’s Nearest Neighbors routine (scikit-learn v1.7.2). For each sampled reference spectrum *i*, we identified *k* nearest hits (same accession) and k nearest misses (different accessions) in the full *P* = 825-dimensional spectral space using Euclidean (L2) distance, and we updated band weights using the absolute per-band difference terms for hits and misses (|*x*_*ij*_ – *x*_*hj*_| and|*x*_*ij*_ – *x*_*rj*_|), as defined in eq (ReliefF). We set the number of nearest neighbors to *k* = 20 and used all training spectra as reference instances (*m* = *n*_*train* = 2560); all randomized operations were controlled by a fixed seed (*random*_*state* = 42) to ensure reproducibility. To avoid information leakage into the independent test set, ReliefF ranking was computed using only the training partition (*n* = 2560) of the fixed stratified 80/20 split.

For the hit search within the same accession, we retrieved *k* + 1 neighbors and excluded the query spectrum itself, retaining the *k* nearest hits.

### 2.8 Classification Modeling Framework

Accession identification was framed as a 32-class classification problem. To ensure a fair comparison across model families, all classical machine learning classifiers and the secondary 1D deep-learning benchmark were evaluated via the same stratified 80:20 hold-out split (training *n* = 2560; test *n* = 640; 80:20 split per accession). Each seed contributed a single ROI-averaged spectrum, preventing any pixel- or seed-level overlap between the training and test sets. We benchmarked 25 classical classifiers spanning decision trees, discriminant analysis, naive Bayes, support vector machines, *k*-nearest neighbors, kernel approximation methods, ensemble learning, and multilayer perceptrons. The primary interpretive emphasis is on the PCA structure, wavelength prioritization, and linear SVM behavior, whereas deep learning is treated as a secondary comparative analysis. Although model predictions are computed at the seed level (*N* = 3200 spectra), seeds are nested within accessions (*G* = 32 accession lots). Therefore, the uncertainty in the performance metrics was quantified via accession-clustered resampling (cluster bootstrap over accessions), and the accession-level (majority-vote) accuracy was treated as a binomial proportion with 32 independent units.

The predicted labels on the independent test set were used to compute the overall accuracy and error rate as follows.

Accuracy=1N∑i=1NoI(yi^=yi)

Errorrate=1−Accuracy

Cab=∑i=1NoI(yi=a)I(yi^=b)

Here, 1 denotes the indicator function, which is equal to 1 when the condition is true and 0 otherwise.

Confusion matrices were computed on the test set and optionally row-normalized for visual comparison of the per-class error structure. For the multiclass precision, recall, and F1-score, class-wise quantities were computed from the confusion matrix and combined via support-weighted averaging; because the data set is balanced (100 seeds per accession), support-weighted and macro-averaged summaries are numerically equivalent. In addition to predictive metrics, training time, prediction throughput (observations s^–1^), and serialized model size were recorded to characterize computational feasibility:

$$\mathrm{Precisio}n_{c}=\frac{{\mathrm{TP}}_{c}}{{\mathrm{TP}}_{c}+{\mathrm{FP}}_{c}}$$

$$\mathrm{Recal}l_{c}=\frac{{\mathrm{TP}}_{c}}{{\mathrm{TP}}_{c}+{\mathrm{FN}}_{c}}$$

$$F1_{c}=\frac{2\;\mathrm{Precisio}n_{c}\;\mathrm{Recal}l_{c}}{\mathrm{Precisio}n_{c}+\mathrm{Recal}l_{c}}$$

$$\mathrm{Precisio}n_{w}=\sum_{c=1}^{C}\left(\frac{n_{c}}{N}\right)\cdot \mathrm{Precisio}n_{c}$$

$$\mathrm{Recal}l_{w}=\sum_{c=1}^{C}\left(\frac{n_{c}}{N}\right)\cdot \mathrm{Recal}l_{c}$$

$$F1_{w}=\sum_{c=1}^{C}\left(\frac{n_{c}}{N}\right)\cdot F1_{c}$$

where TP_*c*_, FP_*c*_, TN_*c*_, and FN_*c*_ denote the true positives, false positives, true negatives, and false negatives for class *c*, respectively. In addition, where *K* = 32 classes, ∑_*c* = 1_^*K*^ *x* is the number of test samples in class *c*, and *N* is the total number of test samples.

We benchmarked 25 classical classifiers to span the major inductive-bias families commonly used in chemometrics and HSIs (linear and nonlinear SVMs, *k*-NN variants, discriminant analysis, naive Bayes, decision trees, and ensemble methods). This model set was intentionally broad to test whether separability is primarily low-dimensional and approximately linear (in which case linear methods should perform competitively) versus requiring stronger nonlinearity or higher model capacity.

### 2.9 Hierarchical Cross-Validation

In addition to the fixed stratified 80/20 training/test split used for primary benchmarking, we performed a genotype-aware hierarchical cross-validation procedure to assess robustness under resampling, while ensuring that all 32 genotypes were represented in every fold. Specifically, we used stratified *K*-fold cross-validation with *K* = 5 applied within each accession, yielding 20 test seeds per genotype per fold (Tables S2 and S3).

### 2.10 Model Optimization Curves and Probabilistic Performance Summaries

To replicate optimization-style diagnostics, two model families were further analyzed via simplified grid evaluations using only the training data and an internal validation split. For *k*-nearest neighbors, the number of neighbors was varied over a finite range constrained by the data set size, and the validation classification error was recorded to identify an appropriate *k*. For multilayer perceptron classification, a discrete set of hidden-layer configurations was evaluated under the same protocol to identify a performant architecture under fixed optimization settings. The independent test set was not used for model selection.

When classifiers provide probabilistic outputs via predict_proba or continuous decision scores via a decision function, score matrices on the independent test set are used to compute micro-averaged receiver operating characteristic (ROC) and precision–recall (PR) curves by binarizing class labels in a one-versus-rest manner and pooling across all classes.^28^ For binary scoring of a positive class, the ROC definitions were as follows:

$$\mathrm{TPR}=\frac{\mathrm{TP}}{\mathrm{TP}+\mathrm{FN}}$$

$$\mathrm{FPR}=\frac{\mathrm{FP}}{\mathrm{FP}+\mathrm{TN}}$$

$$\mathrm{Precision}=\frac{\mathrm{TP}}{\mathrm{TP}+\mathrm{FP}}$$

$$\mathrm{Recall}=\frac{\mathrm{TP}}{\mathrm{TP}+\mathrm{FN}}$$

where TP, FP, TN, and FN denote true/false positives/negatives.

The area under the ROC curve (AUC) and the average precision (AP) were computed as scalar summaries of the ranking quality under the pooled micro-averaged formulation.

### 2.11 Secondary Benchmark: Deep-Learning Models on 1D Spectral Signatures

As a secondary comparison, deep learning was applied directly to the 1D reflectance vectors, treating each spectrum as a fixed-length sequence of length *P* = 825. The ROI-level data set (*N* = 3200) was split into a stratified training set (80%, *n* = 2560) and an independent test set (20%, *n* = 640) reserved exclusively for final evaluation. Within the training set, 15% (*n* = 384) was used for validation during optimization, leaving 2176 spectra for parameter updates. The validation subset was drawn only from the training split, and the independent test set was accessed only once for final reporting, ensuring that reported test metrics reflect generalization to previously unseen seeds. Early stopping (patience of 6 epochs) was monitored via validation loss, and the best-performing model state was restored prior to evaluation on the test set.

The architectures explored ranged from compact and deeper multilayer perceptrons to convolutional and residual 1D models, dilated temporal convolutional networks, recurrent models operating on wavelength-indexed sequences, and transformer encoders with learnable embeddings and multihead self-attention. The models were trained via the Adam optimizer (learning rate 10^–3^), mini-batches of size 32, and a maximum of 25 epochs, with multiclass cross-entropy loss. For a sample *i* with a true label *y*_*i*_ and a predicted probability *p*_*i*, *c*_ for class *c*, the loss was:

$$L=-\frac{1}{N}\overset{N}{\underset{i=1}{\sum }}\log\left(p_{i,y_{i}}\right)$$

The performance of each secondary deep-learning model on the held-out test set was evaluated in terms of accuracy, macro-averaged precision, macro-averaged recall, and macro-averaged F1-score, together with the number of trainable parameters as a measure of model capacity. Training and validation learning curves were recorded for each architecture to document optimization dynamics and detect overfitting behavior under fixed training budgets.

### 2.12 Reproducibility and Figure Generation

All figures, tables, and intermediate outputs reported in this work were generated programmatically from the same Python 3.13.9 workflow used for preprocessing, modeling, and evaluation. Random seeds were fixed across data set splitting and model initialization wherever supported to stabilize comparisons, and performance metrics were derived from the independent 20% test protocol described above, ensuring that reported values reflected generalization beyond fitted data.^44^ All analyses and figure generation were performed in Python 3.13.9 via NumPy 2.2.6, Pandas 2.3.3, Matplotlib 3.10.5, Seaborn 0.13.2, SciPy 1.16.3, scikit-learn 1.7.2, and openpyxl 3.1.5. The deep learning models were implemented in Keras 3.11.3 with TensorFlow 2.20.0. All random operations (data splitting, model initialization, and bootstrap resampling) used fixed random seeds to ensure reproducibility.

Model development and computational routines were executed on a workstation equipped with an NVIDIA GeForce RTX 4090 GPU, an Intel Core i9-14900HX CPU, 128 GB RAM, and NVMe storage, supporting efficient handling of high-dimensional spectral data sets and enabling consistent benchmarking across classical and deep learning approaches.^17,44^

## 3 Results

### 3.1 Phenotypic Diversity and Accession Benchmarks

The benchmark set comprised thirty-two grain-legume accessions (A01–A32) selected to span a wide phenotypic space in terms of seed coat color, patterning, and grain morphology, thereby creating a demanding authentication scenario under controlled laboratory acquisition (Figure 1 and Table S1). The collection included uniformly colored seed coats across high-albedo (white/cream), intermediate (yellow, pink, and red), and low-albedo (brown to black) phenotypes, as well as heterogeneous patterns such as mottling, striping, and bicolour hilum contrast. This visual diversity was accompanied by substantial variation in grain geometry, ranging from small, rounded seeds to large kidney-shaped and elongated “peanut-type” morphologies. Two non-*Phaseolus* accessions (A27 and A28) were deliberately included to expand the explored phenotypic manifold beyond common bean while remaining within the local “Feijões” classification, thereby increasing the practical relevance of the benchmark for real-world screening contexts where mixed lots may occur. A total of 3200 ROI-level spectra were analyzed, with 100 seeds per accession (Figure 2), enabling both population-level spectral characterization and robust model benchmarking across visually separable and visually ambiguous cases.

**Figure 1:**
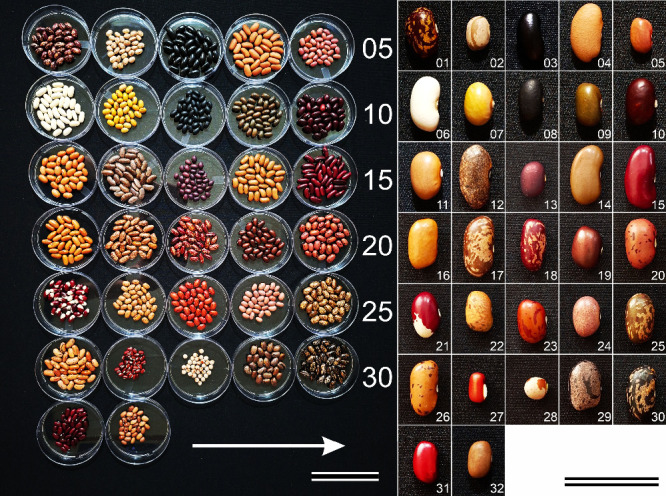
Seed lots from 32 grain-legume accessions were prepared for VNIR hyperspectral image acquisition via a Headwall system. The left panel shows 10 cm Petri dishes arranged sequentially from A01 to A32 from left to right within each row (arrow), with the sequence restarting at the left edge of the subsequent row; the numerals at the right indicate the final accession in each row. The right panel shows one representative seed per accession (01–32) at a common magnification to provide a reference for seed coat color, patterning, and morphology. Accessions A01–A26 and A29–A32 are *Phaseolus vulgaris* L.; A27 is *Vigna angularis* (Willd.) Ohwi and H. Ohashi and A28 is *Cajanus cajan* (L.) Huth. Scale bars: 8.5 cm (left) and 3 cm (right).

**Figure 2:**
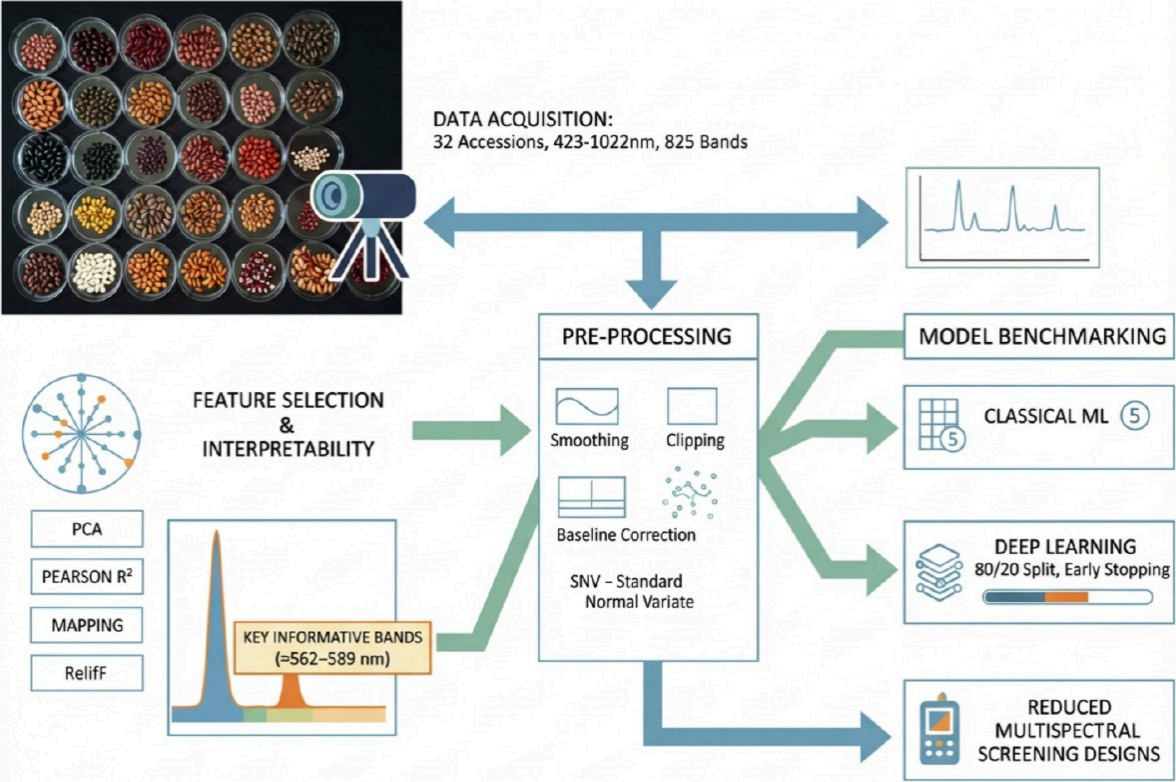
Workflow for full-spectrum VNIR hyperspectral seed accession identification based on ROI-averaged 1D reflectance spectra and interpretable modeling. Seeds from 32 grain-legume accessions were scanned with a Headwall push-broom system (≈423.34–1022.07 nm; 825 bands). After radiometric calibration (dark and Spectralon white reference), one mean 1D spectrum was extracted per seed from a central 10 × 10-pixel ROI (*N* = 3200). The spectra were preprocessed (Savitzky–Golay smoothing; clipping to [0, 1]; optional baseline correction and SNV), and interpretability analyses (PCA, one-versus-rest Pearson *r* ^2^ association mapping, and ReliefF ranking) were performed. Classical machine-learning models and the secondary 1D deep-learning benchmark were evaluated under the same stratified 80/20 training/test split, with an internal validation set and early stopping. ReliefF revealed a compact region of maximally informative bands concentrated in the green domain (≈562.85–584.65 nm), providing direct guidance for reduced-band multispectral screening designs, whereas all the predictive benchmarks reported here used the complete 825-band spectra. The reduced-band sensitivity results are reported in Table S4.

### 3.2 Accession-Level Reflectance Organization across the VNIR Range

The accession-averaged reflectance profiles exhibited strong and structured differences across the measured VNIR axis (Figure 3a). In the visible bands, the reflectance remained relatively low and accession dependent, which is consistent with variable visible absorption (color-related) and coat microstructures altering both the baseline albedo and the wavelength-specific attenuation. A pronounced transition occurred around the visible-to-near-infrared boundary, after which the reflectance increased and formed a broad near-infrared plateau. This behavior is expected because reflectance transitions from strong visible absorption to higher, smoother near-infrared reflectance that is primarily shaped by scattering as the wavelength increases beyond ≈700 nm.

**Figure 3:**
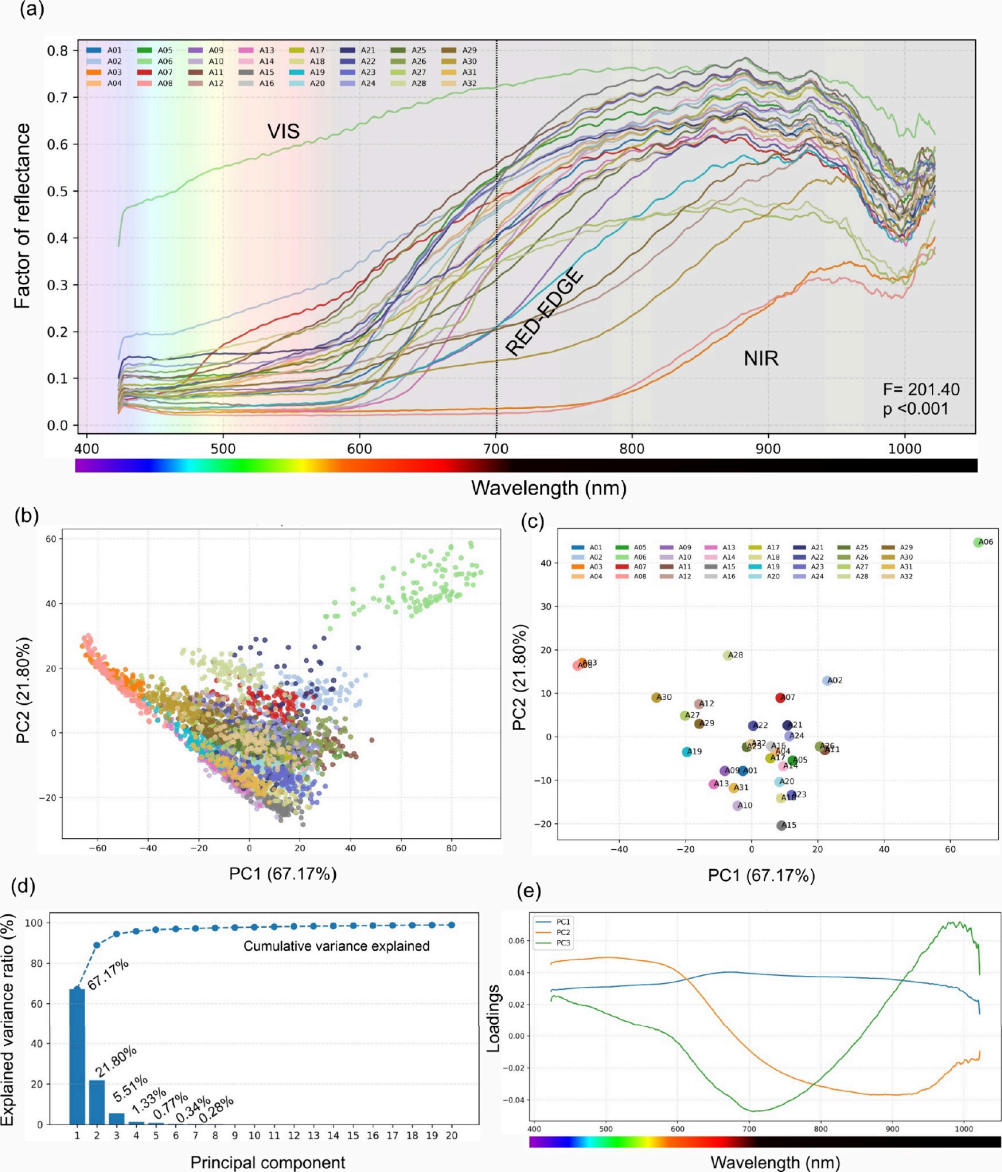
VNIR reflectance signatures and PCA-derived variance organization for the 32 grain-legume accessions measured with the Headwall hyperspectral system (≈423–1022 nm; 825 bands). (a) Accession-level mean reflectance profiles across the visible and near-infrared domains; the dashed line marks 700 nm, and PERMANOVA on the spectral distance matrix confirms a strong accession effect (*F* = 201.40, *p* < 0.001). (b) PCA score distributions for all the ROI spectra projected onto PC1 and PC2 (PC1 = 67.17%, PC2 = 21.80%), colored by accession. (c) The same PC space is shown as accession centroids to emphasize class-level separation. (d) Explained variance for the first 20 components with cumulative variance overlaid. (e) Loading vectors for PC1–PC3, indicating the wavelength regions contributing most strongly to each variance mode.

Although the overall shape was conserved, the magnitude and curvature of this transition and the subsequent plateau differed substantially among accessions, yielding high separability in multivariate space. The white, high-albedo accession A06 showed systematically elevated reflectance throughout the spectrum, whereas darker phenotypes retained lower visible reflectance and a damped transition profile. PERMANOVA of the spectral distance matrix confirmed a strong accession effect (*F* = 201.40, *p* < 0.001; Figure 3a), indicating that the observed differences were not attributable to sampling variability within accessions.

### 3.3 PCA Reveals a Low-Dimensional Structure Linked to Broad-Band Absorption and Scattering Regimes

Principal component analysis compressed the 825-dimensional spectra into a low-dimensional representation in which a small number of variance modes captured most of the observed spectral diversity (Figure 3b–e). PC1 alone accounted for 67.17% of the variance, with PC2 contributing an additional 21.80% and PC3 contributing 5.51%, such that the first three components explained 94.48% of the spectral variability. In the ROI-level score space, spectra formed a continuous wedge-shaped manifold rather than strictly separated clusters, which is consistent with a scenario in which accession identity is encoded by graded shifts in underlying optical properties rather than by a set of discrete, nonoverlapping signatures. Despite this continuity, the accession centroids are clearly separated in the PC1–PC2 plane (Figure 3c), with A06 occupying an extreme region with large positive scores, reflecting its globally elevated reflectance. Several accessions were displaced toward strongly negative PC1 values, indicating a contrasting regime characterized by suppressed reflectance across the spectrum and/or a weaker visible-to-NIR transition. The PCA loading profiles revealed that these variance modes were driven by a wavelength-contiguous structure, supporting a mechanistic interpretation in which the dominant axes correspond to broad optical regimes, such as whole-spectrum albedo and the balance between visible absorption and NIR scattering, rather than to isolated single-band artifacts ( Figure 3e).

### 3.4 Wavelength-Resolved Associations and Band Prioritization Highlight a Narrow Window of Green Discrimination

One-versus-rest association mapping demonstrated that the accession-related signal was unevenly distributed across the VNIR axis (Figure 4a,b). The Pearson association profiles revealed accession-specific sign patterns and wavelength-dependent magnitudes, indicating that different accessions are distinguished by different portions of the spectrum rather than by a single universal band. When expressed as coefficients of determination (*r* ^2^), the association structure emphasized localized wavelength regions that carried strong membership-linked signals for specific accessions. The strongest single association across the complete accession–wavelength map reached *r* ^2^ = 0.736 at 424.79 nm, indicating that, for at least one accession, a narrow blue/violet region alone can encode a substantial fraction of the one-versus-rest variance.

**Figure 4:**
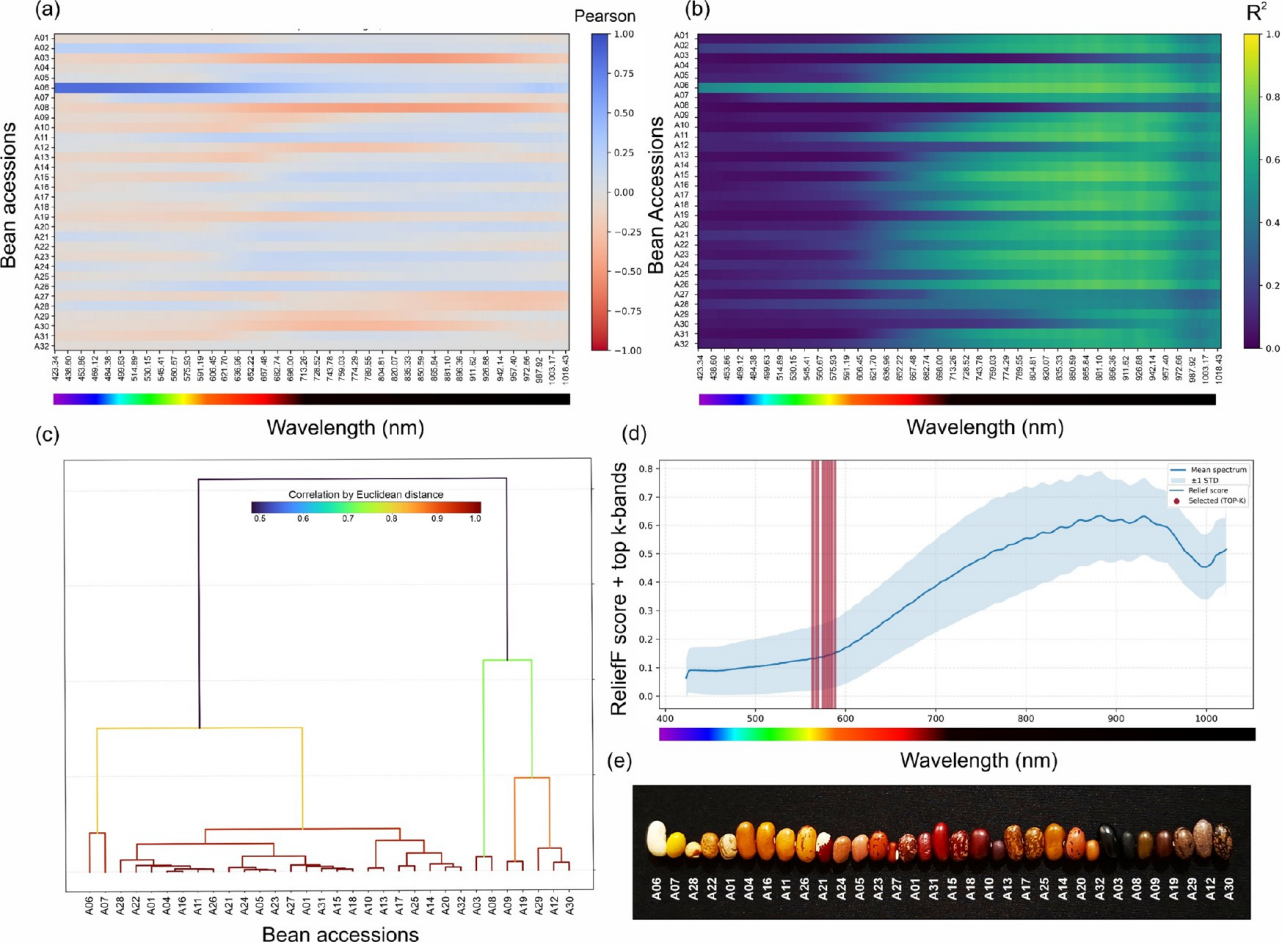
Wavelength-resolved association mapping, accession similarity structure, and supervised band ranking from the Headwall VNIR spectra. (a) Pearson association (*r*) between reflectance at each wavelength and accession membership under one-versus-rest encoding, shown for all 32 accessions across the measured spectral axis. (b) The same association map expressed as *r* ^2^ to emphasize wavelength regions carrying the strongest class-linked signal. (c) Hierarchical clustering of accession-mean spectra on the basis of Euclidean distance, defining a spectral neighborhood structure among accessions. (d) ReliefF importance profile across wavelengths, shown with the global mean spectrum and ±1 SD envelope; vertical markers indicate the selected top-*K* bands (*K* = 16), concentrated in a narrow green window (≈562.85–584.65 nm). (e) Representative seeds ordered to match the dendrogram leaf order, linking spectral neighborhoods to seed coat phenotypes.

The spectral neighborhood structure derived from distances between accession-mean spectra yielded a hierarchical organization in which certain accessions formed tightly coupled pairs, whereas others occupied more isolated branches (Figure 4c). Notably, supervised band ranking via ReliefF converged on a compact and practically deployable set of informative wavelengths concentrated in the green domain: the top-ranked bands spanned 562.85–584.65 nm, with peak importance centered at approximately ≈582 nm (Figure 4d). This result provides a nontrivial design insight: whereas the strongest single-class associations may occur at shorter wavelengths, the multiclass discrimination signal shows its highest local concentration in this narrow green window; however, reduced-band sensitivity tests indicate that this window alone is insufficient for the full 32-class task, implying that complementary information is distributed across the VNIR range. The narrow green window is consistent with the notion that subtle differences in visible-domain seed coat appearance (color/pattern) and coat scattering produce maximal between-accession contrast where the reflectance transitions between absorption-dominated and scattering-dominated regimes. The representative seed ordering along the dendrogram leaves further illustrates that spectrally proximal accessions often share gross visual characteristics, implying that spectral similarity and phenotypic similarity are coupled but not identical (Figure 4e).

### 3.5 Multiclass Accession Classification: Strong Performance with Concentrated Failure Modes

Multiclass classification using the full VNIR spectra produced a clear hierarchy across the evaluated classical models (Figure 5). The overall test-set accuracy ranged from 11.56% (fine Gaussian SVM) to 88.59% for the linear SVM, with several shallow neural network classifiers and discriminant variants performing comparably in the high-accuracy tier. The linear SVM achieved the best overall performance (accuracy = 88.59%; weighted F1-score = 88.52%) and the lowest total misclassification count (73 errors on the 640-sample test split), indicating that a linear decision boundary in the high-dimensional spectral space is sufficient to exploit the dominant discriminative structure present in these data (Figures 5, S1, and S2).

**Figure 5:**
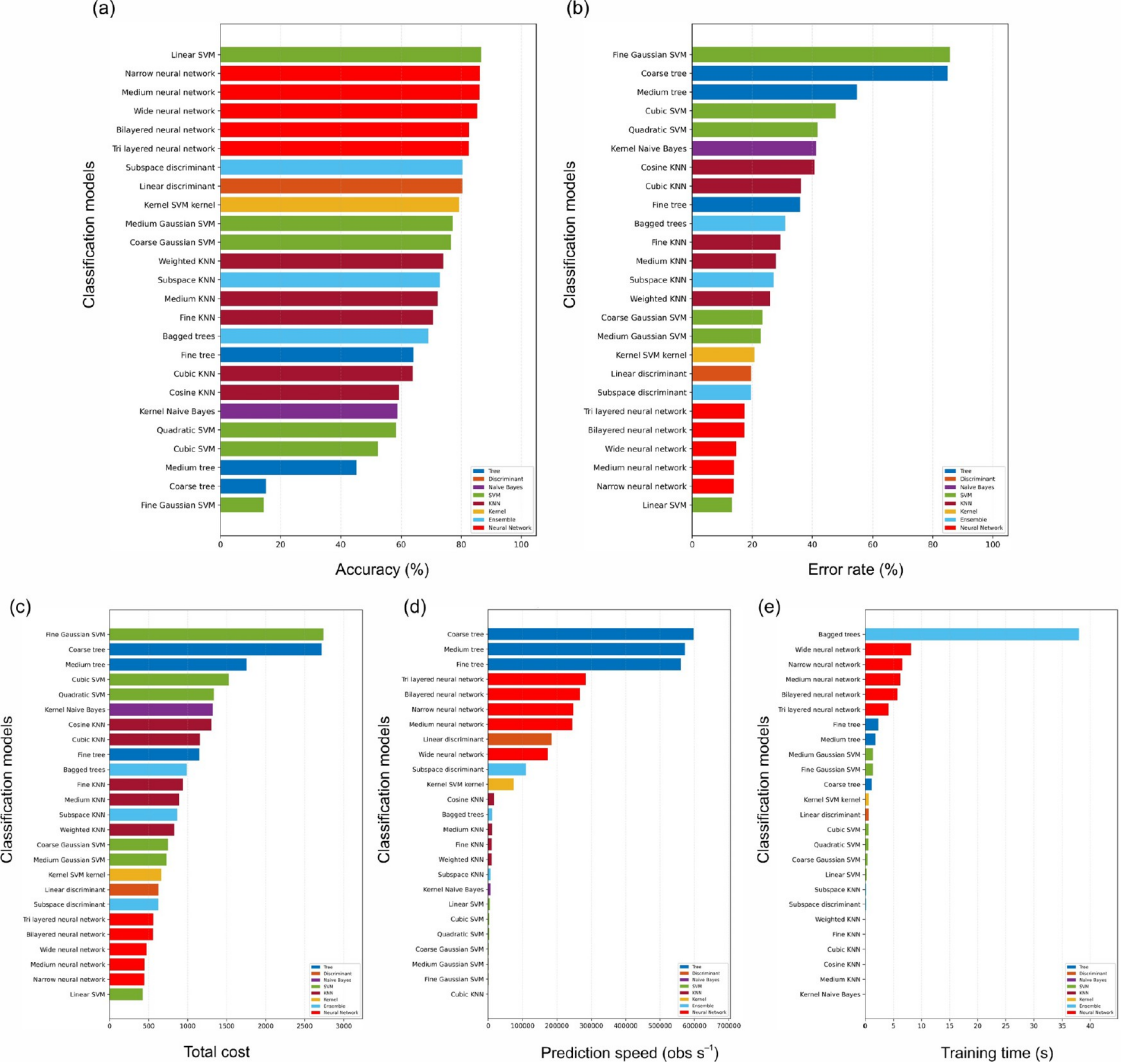
Multiclass accession classification benchmarking and computational profiling using the complete VNIR spectra. (a) test-set accuracy and (b) test error rate, alongside deployment-relevant diagnostics including (c) total misclassification cost, (d) prediction throughput (observations s^–1^), and (e) training time (s), highlighting performance–efficiency trade-offs across the evaluated classifier families.

At the seed level, the best classical model achieved 88.59% accuracy (accession-clustered bootstrap 95% CI: 84.53–92.34%) and an 88.52% weighted F1-score (95% CI: 85.88–91.20%) for the linear SVM, whereas the best secondary 1D deep-learning benchmark (MLP_1D) achieved 82.03% accuracy (95% CI: 75.94–87.66%) and an 81.52% weighted F1-score (95% CI: 78.59–89.76%). The selected classical baseline metrics are summarized in Tables S2 and S3, and the broader benchmark distribution is shown in Figures 5 and S1. Reduced-band sensitivity tests (Table S4) revealed that restricting features to the ReliefF top-16 wavelengths or to the green window alone markedly reduced performance (Seed Acc ≈ 32–34%), whereas uniform down-sampling across the full VNIR range retained high accuracy (e.g., 89.06% at 5 nm and 87.50% at 10 nm). At the accession (line) level, the majority of the accessions across the 20 test seeds per accession yielded 100% accuracy for the linear SVM (32/32 accessions; exact 95% CI: 89.10–100%) (Table S2) and 96.88% accuracy for the best deep model (31/32; exact 95% CI: 83.81–99.86%).

The confusion matrices were strongly diagonal overall, but the residual errors were not uniformly distributed; instead, they were concentrated into a small number of consistent near-neighbor confusions (Figure 6). For the linear SVM, the largest within-class confusion was A12 → A30 = 25% (5/20), with the reciprocal confusion A30 → A12 = 15% (3/20). Additional structured confusion included A14 ↔ A17 = 15% in both directions (3/20 each), A03 → A08 = 15% and A08 → A03 = 10%, and a cluster involving A11, A04, and A26 (e.g., A11 → A04 = 15%, A04 → A11 = 10%, A11 → A26 = 15%, A26 → A11 = 10%), further indicating that certain accessions occupy closely overlapping VNIR regimes despite distinct labels. Importantly, these high-frequency confusions align with the spectral neighborhood structure inferred from distance-based clustering, which is consistent with an interpretation in which classification errors primarily arise from genuinely similar mean reflectance profiles rather than from stochastic model instability (Figures 4c, 6, and S3).

**Figure 6:**
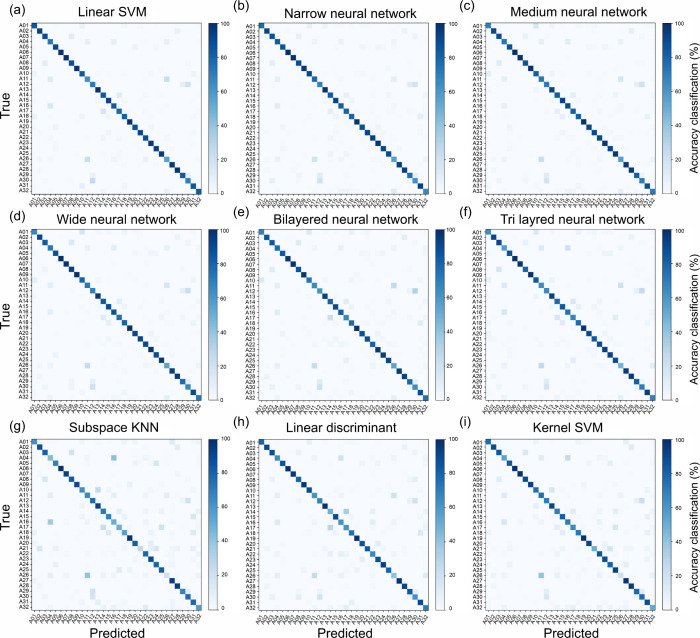
Confusion matrices for selected classical classifiers applied to the 32-accession discrimination task. Panels show class-conditional prediction outcomes (true labels on the *y*-axis; predicted labels on the *x*-axis) for representative models, including linear SVM, neural network variants, subspace KNN, discriminant analysis, and a kernel SVM. The color intensity denotes the within-class normalized frequency; diagonal dominance indicates robust recognition, whereas the off-diagonal structure indicates systematic confusion among spectrally adjacent accessions.

The mean spectra and corresponding difference spectra for the most frequently confused accession pairs are provided in Figure S3, and PCA score plots highlighting only the confused test-set seeds (with representative seed images) are provided in Figure S4.

### 3.6 Secondary 1D Deep Learning Benchmark

As a secondary comparison, deep learning models trained directly on the 1D VNIR reflectance sequences showed architecture-dependent learning dynamics and generalization (Table 1 and Figures 7 and 8). The best held-out performance was achieved by MLP_1D (accuracy = 82.03%; F1-score = 81.52%), followed by MLP_Wide (accuracy = 80.94%; F1-score = 80.06%) and MLP_Deep (accuracy = 79.69%; F1-score = 78.53%). The remaining architectures were clearly below the linear SVM benchmark reported above, indicating that, under this ROI-averaged and moderate-size data set, the dominant discriminative structure is more effectively captured by the classical models.

**Table 1:** Independent Test-Set Performance of the Secondary One-Dimensional Deep-Learning Architectures for Classifying the 32 Grain-Legume Seed Accessions from Headwall VNIR Hyperspectral Reflectance Sequences (≈423–1022 nm; 825 Bands)^a^

**model**	**accuracy**	**F1-score**	**precision**	**recall**	**parameters**	**epochs**
MLP_1D	82.031	81.521	83.106	82.031	116,064	25
MLP_Wide	80.938	80.058	82.239	80.938	439,328	20
MLP_Deep	79.688	78.533	82.610	79.688	254,688	25
Transformer_1D	62.813	60.603	64.770	62.813	69,152	23
CNN1D_Deep	55.781	53.723	59.493	55.781	57,536	25
ResNet1D	53.125	50.568	57.610	53.125	77,600	25
TCN1D	50.313	47.314	51.703	50.313	77,344	25
Inception1D	46.094	42.680	45.448	46.094	19,504	23
CNN1D	39.688	35.825	38.646	39.688	12,832	25
BiLSTM1D	26.406	21.288	24.648	26.406	38,432	13

a Accuracy is reported as the overall percentage (%) of correctly classified test spectra; precision (%), recall (%), and F1-score (%) summarize multiclass performance over the full accession set. “Parameters” denotes the number of trainable weights in each network. “Epochs” indicates the epoch at which training terminated under early stopping (maximum of 25 epochs) (Figure 7).

**Figure 7:**
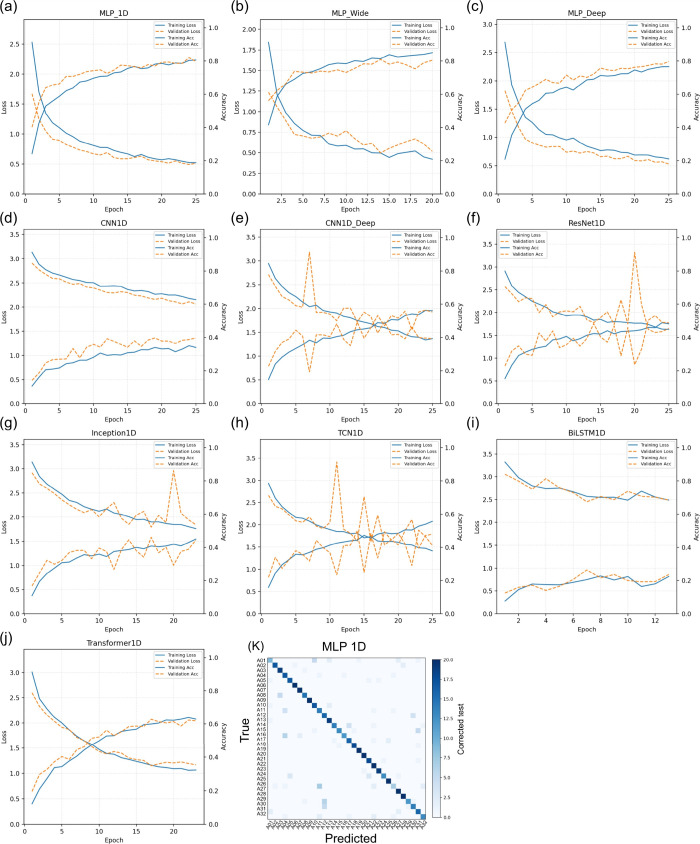
Training dynamics of the secondary one-dimensional deep learning benchmark fitted directly to VNIR reflectance sequences. Panels (a–j) show epoch-wise training and validation loss and accuracy traces for the evaluated architectures. Panel (*k*) presents the confusion matrix on the independent test split for the best-performing deep model (MLP_1D), summarizing residual error structure after optimization.

**Figure 8:**
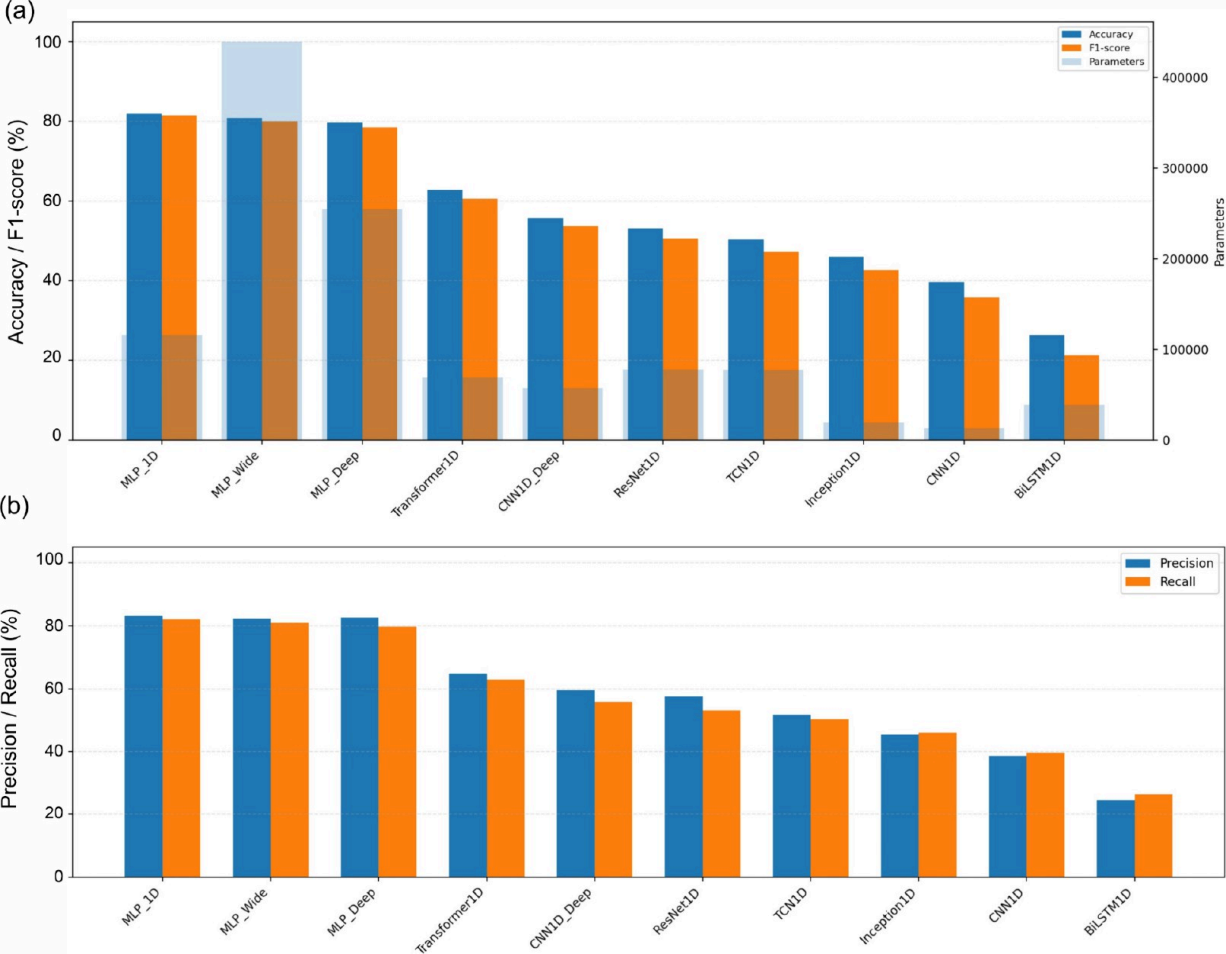
Test-set performance and capacity comparison across the secondary deep learning models for accession classification. (a) Accuracy and F1-score are shown for each architecture and are jointly displayed with the model size (number of trainable parameters; secondary axis) to visualize the performance–complexity relationships. (b) Precision and recall for the same models.

The optimization traces were consistent with this ordering (Table 1 and Figure 8). The MLP family converged more stably than the other 1D deep networks, but no architecture exceeded the primary linear SVM benchmark. In parallel optimization diagnostics, KNN tuning identified an optimal neighborhood size of *k* = 6 and yielded micro-averaged AUC = 96.40% and AP = 76.20%, whereas the tuned shallow neural network classifier achieved micro-averaged AUC = 99.70% and AP = 93.70% (Figure S2). Thus, the probabilistic ranking quality remained high even when discrete accuracy was limited by a small number of spectrally overlapping class pairs (Figures 6 and S3–S5).

## 4 Discussion

### 4.1 Spectral Heterogeneity Across Accessions and Its Chemical Plausibility

The measured range (423–1022 nm; 825 bands) intentionally spans the visible domain, where testa color and pattern are expressed optically, and the short near-infrared region, where weaker overtone structure and path-length effects can affect the hydration state and microstructural organization. This choice is consistent with how recent seed-oriented hyperspectral pipelines position VIS–NIR acquisition as a compromise between biological information usefulness and sensor practicality. In these pipelines, the visible bands typically drive discrimination for testa color- and surface-mediated phenotypes, while the near-infrared bands act as complementary support for composition- or moisture-related variation.^9,11,32,45^ In the present panel, the mean preprocessed spectra indicate that separability between accessions is indeed dominated by the visible region, matching the morphological diversity of testa color and patterning (Table S1). This is not merely descriptive: it aligns with the expectation that testa optics are governed by visible absorption and wavelength-dependent scattering. Accession-level differences in seed coat color and patterning, together with surface microstructures, should produce stable and interpretable spectral signatures.^2,32^ Framed in this way, hyperspectral phenotyping becomes most informative when spectral variance can be traced to reproducible phenotype-generating mechanisms rather than treated as an abstract label space.^2,32^

The internal consistency of this interpretation is reinforced by the PCA. The variance is concentrated in a small number of directions and repeatedly highlights the same wavelength regions, with dominant loadings placing PC1 in the red (≈667–681 nm), PC2 in the blue–green (≈503–507 nm), and a subsequent component in the water-sensitive near-infrared region (≈983–995 nm) (Figure 3). Mechanistically, this structure is coherent with the broader literature, in which visible components primarily capture visible absorption (color-related). In addition, scattering-driven slope changes, whereas near-infrared components capture weaker O–H-related signatures and effective optical path-length changes linked to the internal microstructure and residual water.^9,13,22^ Importantly, PCA is therefore not used here for generic compression; it serves as an interpretive bridge between sensor output and biological plausibility, indicating that discrimination is predominantly driven by visible-domain absorption and surface scattering, whereas near-infrared information contributes more subtly and may matter most when accessions are visually convergent.^32,45^

### 4.2 PCA as a Mechanistic Bridge between Spectral Variance and Screening Efficiency

The observation that the first three principal components explain 67.17, 21.80, and 5.51% of the variance implies that most spectral variability lies on a smooth, low-dimensional manifold. In chemometric terms, this is the expected consequence of wavelength-contiguous redundancy: adjacent bands are strongly correlated, and physical absorption/scattering processes generate smooth structures rather than arbitrary high-frequency variations. Recent perspectives at the interface of hyperspectral imaging and chemometrics emphasize that this low intrinsic dimensionality is precisely why parsimonious linear methods remain competitive for many spectral tasks,^21,45,46^ particularly when spatial information is collapsed, and the dominant signal is the global reflectance shape.^5,23^ In screening terms, the same geometry provides a mechanistic reason why the strongest classical model is also among the simplest: the dominant structure can be separated by a linear margin without requiring a high-capacity decision function. Consistent with this, the linear SVM yields the highest overall accuracy (88.59% across 32 balanced classes; Figure 5) while remaining computationally efficient, aligning model choice with process constraints in high-throughput phenotyping and breeding workflows.^32,40^

A key added value of PCA in this study is that it supports operational forecasting of classification difficulty before the classifier is trained. Accessions that occupy crowded neighborhoods in PC space exhibit the strongest confusion in supervised classification (Figure 6), whereas more isolated accessions tend to be recognized with higher class-conditional accuracy. This observation connects directly to recent arguments that model evaluation in hyperspectral pipelines should be coupled with data geometry and representational diagnostics rather than relying solely on aggregate metrics. This is important because many failure modes arise from intrinsic overlap rather than optimization instability.^10,46^ The agreement between the unsupervised structure (correlation/clustering; Figure 4a–c) and supervised errors suggests that misclassifications reflect overlapping optical phenotypes and therefore have biological meaning.^5,32^ They are signatures of convergent seed coat color and microstructure configurations rather than artifacts of the modeling pipeline.^21,45,46^

### 4.3 What Model Ranking Reveals the Nature of the Signal?

Model ranking provides additional mechanistic insight because performance is consistent with a smooth, redundant signal in which linear or shallow nonlinear decision functions capture most of the discriminative structure, whereas deeper architectures offer limited gains under the current representation and sample size ( Figures 5–8). This pattern matches recent comparative analyses showing that deep learning tends to outperform classical chemometrics mainly when it can exploit a rich spatial-spectral structure or much larger training diversity. For mean-spectrum representations with high collinearity, classical margins and low-rank projections often remain highly competitive.^5,23^ The present results therefore reinforce the primary emphasis on PCA, interpretable wavelength structure, and linear SVM classification in this data set.

Within the secondary deep-learning subset, MLP_1D performed best (accuracy = 82.03%), followed by MLP_Wide (80.94%), whereas more structured sequence architectures, including the transformer variant, did not close the gap to the linear SVM (Figures 7 and 8; Table 1). This suggests that representation, not only architecture, constrains what can be learned here. By averaging spectra across the seed object, the pipeline intentionally removes local textural and pattern information that may carry diagnostic scattering cues for mottled or striped testas. Approaches that retain spatial heterogeneity or perform pixel-level spectral unmixing can therefore be viewed as mechanistically motivated extensions for future work rather than as the present analytical focus.^21,32,45–47^

A further point of convergence with the seed-focused literature emerges in the visible domain. Recent reviews and crop-specific studies report that blue-green wavelengths often carry strong leverage for discriminating testa-driven phenotypes. Our data indicate that the strong PC2 loading at approximately 503–507 nm is consistent with differences in visible seed coat coloration, i.e., wavelength-dependent absorption/scattering in the blue-green region.^32,45^ The green discrimination window identified by ReliefF (562.85–584.65 nm) is therefore interpreted as an optical leverage region linked to seed coat color/pattern and surface microstructure, where modest pigment-related absorption differences and wavelength-dependent scattering can produce comparatively large reflectance contrasts. However, no targeted chemical assays or pigment quantification were performed here, so this remains an optically grounded hypothesis rather than a direct chemical attribution.

In related seed and bean classification studies, the reported accuracies vary widely with the number of classes, acquisition conditions, and evaluation protocol. For example, Li et al.^11^ reported 93.06% accuracy for common bean variety identification via an improved ResCNN trained on 66 selected wavelengths with augmentation and SMOTE, whereas Zhu et al.^9^ reported 91.25% accuracy via full-spectrum PLS-DA and 88.75% accuracy via a reduced set of 93 wavelengths (MC-UVE-BOSS) for maize seed aging discrimination. Although these studies address different targets and protocols, they support the broader observation that substantial performance can often be retained under reduced-band designs—motivating our reduced-band sensitivity analysis (Table S4).

### 4.4 From Discriminative Wavelengths to Sensor Design

Relief-ranked features are concentrated in a narrow window (562.85–584.65 nm; Figure 4d), implying that discriminative information is encoded primarily in subtle chromatic transitions rather than broad-band signatures. Mechanistically, this interval lies on a visible-domain slope where pigment-related absorption gives way to rising reflectance toward the yellow-orange region, so small differences in seed coat pigmentation, pattern expression, and microstructural scattering can modulate reflectance in a stable, repeatable manner. The fact that the information localizes rather than spreads diffusely across the spectrum aligns with the modern band-selection literature, which increasingly frames wavelength selection as the identification of phenotype-linked leverage regions that preserve separability while reducing redundancy and computational burden.^32,35,48^ A useful comparative angle is that when the task shifts from accession discrimination toward compositional or quality attributes, near-infrared structure often becomes more dominant, so the visible localization observed here is itself informative about the nature of the underlying signal, namely, seed coat optics rather than bulk chemical composition.^8,13,22^ Notably, our reduced-band tests (Table S4) indicate that restricting the input to this narrow window alone does not preserve the full 32-class accuracy, implying that complementary information across the VNIR range remains necessary.

This localization also has direct translational implications for sensor engineering. Recent work on hand-held and real-time spectroscopy in food and agricultural monitoring emphasizes that deployability depends on an explicit trade-off between spectral richness, hardware complexity, and decision reliability; in that context, a narrow candidate window offers a credible path from hyperspectral discovery to compact multispectral deployment.^13,14^ A two-stage strategy therefore follows mechanistically rather than heuristically: first, full hyperspectral acquisition is used to identify and validate phenotype-linked windows under controlled conditions, then those windows are distilled into a reduced design, and the models are retrained under the intended operational constraints, with explicit stress testing against nuisance variation.^5,10,32^ In this framing, sensor reduction is successful only if it preserves sensitivity to phenotype-generating optics while suppressing spurious variability arising from acquisition conditions, aligning with recent calls for robust, deployment-aware hyperspectral pipelines in digital agriculture.^10,28,39,49,50^

### 4.5 Moving Beyond Correlation: A Causal–Probabilistic Interpretation of Spectra

Most hyperspectral classification pipelines remain correlational in the sense that they identify discriminative patterns without explicitly modeling the mechanisms that generate them. The present results nevertheless contain three convergent signals that support a stronger, testable mechanistic account: the PCA geometry, the structured confusion pattern, and the localization of discriminative wavelengths. Together, they imply a plausible generative chain in which accession-level genetic and developmental differences shape the seed coat composition and coat microstructure, which then determine visible reflectance and, more weakly, near-infrared absorption, whereas the classifier approximates inverse mapping from spectra to accession identity. This interpretation is compatible with modern seed phenotyping work that treats spectral signatures as mechanistically anchored phenotypes and seeks to connect discriminative regions to physically interpretable optical changes.^11,32,45^ This finding is also consistent with genetic evidence that seed coat color and patterning in common bean, maize, rice, and tomato are regulated by identifiable transcriptional programs and can shift with environmental modulation, providing a biological substrate for both stability and controlled plasticity in spectral signatures.^2^

Turning this mechanistic narrative into credible causal claims requires more than adding model complexity; it requires a measurement-inference framework that remains valid under perturbation. Recent surveys in causal machine learning stress that credible causal reasoning depends on explicit assumptions, invariance principles, or interventional designs, and position papers in digital agriculture similarly converge on causality, explainability, and uncertainty as joint requirements for trustworthy decision systems.^39,42^ This is especially relevant here because illumination shifts, sensor drift, storage effects, and seed moisture differences can induce spectral changes that are observationally indistinguishable from biological differences unless data collection explicitly separates these factors.^10,13^ In parallel, spectroscopy-focused reviews argue that explanations should be treated as physical consistency checks, suggesting a concrete role for interpretability methods: to test whether highlighted wavelengths and learned decision cues remain stable under controlled perturbations that should not affect intrinsic seed coat composition.^16,30^ Within this causal framing, probabilistic modeling becomes functional because calibrated uncertainty can be used to detect spectrally ambiguous cases in dense neighborhoods and trigger remeasurement, complementary assays, or targeted acquisition. This links inference outputs to operational decisions in high-throughput workflows.^14,27,39,42,46^

### 4.6 Limitations and Implications for Translational Deployment

Interpretation is constrained by the chosen representation and by the external validity assumptions. The mean spectra efficiently capture global testa reflectance behavior, but they suppress spatial cues that may matter for mottled or striped accessions and reduce sensitivity to embryonic viability and texture-dependent scattering. This limitation is consistent with the observation that the most difficult accessions occupy crowded regions of PC space and are responsible for disproportionate confusion, implying that the missing information is plausibly spatial rather than purely spectral. Recent seed and crop hyperspectral literature consistently shows that the strongest gains from advanced architectures arise when the spatial-spectral context is preserved, which reinforces the conclusion that future improvements should target representation rather than simply increasing model capacity.^21,45,46^ External validity also remains to be stress tested across acquisition days, instruments, storage conditions, seed moisture levels, and harvest histories. Because only one seed lot was analyzed per accession, the present benchmark does not isolate how these factors reshape VNIR signatures, and robust deployment will require explicit cross-domain evaluation rather than within-domain metrics alone.^10,35,46^

Despite these constraints, the translational message is mechanistically clear. The dominant discriminative information is concentrated in a visible-dominated, low-dimensional structure that can be exploited efficiently via linear margins and carefully distributed wavelength sampling, which aligns naturally with high-throughput screening needs in breeding, certification, and authentication pipelines.^32,40^ The most direct route to stronger mechanistic inference is to convert the empirically identified wavelength regions into validated optical proxies by pairing them with targeted chemical assays of the seed coat constituents. This can be further strengthened by adopting causal and explainable frameworks that separate biological signals from measurement artifacts under controlled perturbations.^8,30,39,42^ This shift moves hyperspectral seed analysis from accurate but potentially fragile classification to interpretable discrimination, in which spectral features map onto measurable mechanisms and uncertainty actively guides decision-making in high-throughput, real-world workflows.^13,14^

A key limitation is that spectral interpretations are not directly validated by targeted chemical assays. Therefore, mechanistic statements are framed as optically plausible hypotheses supported by the literature and by the smooth, wavelength-contiguous variance structure observed here. We did not perform chemical, pigment, or microstructural quantification; therefore, the interpretation of the green window is limited to optical plausibility rather than direct biochemical confirmation. Direct validation assays were outside the scope of the present nondestructive study but are planned for follow-up work.

## 5 Conclusions

The VNIR hyperspectral reflectance (≈423–1,022 nm; 825 bands) enabled accurate seed-level discrimination of a diverse panel of 32 grain-legume accessions (*N* = 3,200 spectra). Under a stratified 80/20 hold-out split (20 test seeds per accession), the best classical model, a linear SVM, achieved 88.59% seed-level accuracy and 100% line-level (accession-level majority-vote) accuracy, whereas the best secondary 1D deep-learning benchmark (MLP_1D) reached 82.03% seed-level accuracy. The error patterns were structured and concentrated among a small set of optically similar accessions, which was consistent with a genuine overlap in the mean reflectance profiles. Exploratory PCA and band-importance analyses indicate that separability is largely governed by smooth, wavelength-contiguous spectral-shape variation, with importance peaking in a narrow green interval (≈562.85–584.65 nm). However, reduced-band sensitivity tests show that restricting classification to that interval alone is insufficient for the full 32-class task, whereas evenly distributed bands across the VNIR range can retain high accuracy with a substantially reduced number of bands. This supports practical multispectral designs that distribute bands across the VNIR range while prioritizing high-information regions. Key limitations are (i) the absence of targeted chemical/pigment/microstructural assays to validate mechanistic interpretations beyond optical plausibility, (ii) acquisition under controlled conditions via a single instrument, and (iii) the use of one seed lot per accession, so robustness to storage conditions, seed moisture content, harvest time, batch-to-batch variability, and instrument drift remains to be stress-tested. Future work should (i) evaluate robustness across storage periods, moisture states, harvest histories, and acquisition batches via hierarchical evaluation designs, (ii) optimize and validate reduced-band multispectral configurations (band placement and count) explicitly for deployment constraints, and (iii) integrate spatial-spectral modeling to better capture heterogeneity in patterned (mottled/striped) accessions.

## Supplementary Material



## Notes

Additional experimental details, bean accession information, hyperspectral image acquisition and preprocessing parameters, model training and validation details in supplementary figures and tables; Figures S1–S5: Classifier benchmarking, KNN and neural-network hyperparameter-optimization traces, confused-accession spectral and PCA analyses with representative seed images, and accession-clustered bootstrap accuracy distributions; Tables S1–S4: Accession list and seed phenotype descriptions, hold-out and K-fold cross-validation performance summaries, and spectral band-selection comparison (PDF)

## References

[1] Aguilar C. L., de Carvalho Júnior O. A., de Carvalho O. L. F. (2025). Decoding
Brazil’s bean belt: spatiotemporal patterns, production systems
and the pulse of bean production (2011–2022). Geo: Geogr. Environ..

[2] Parker T., Bolt T., Williams T., Penmetsa R. V., Mulube M., Celebioglu B. (2024). Seed color patterns in domesticated common
bean are regulated by MYB-bHLH-WD40 transcription factors and temperature. Plant J..

[3] de
Paula E., Almeida R. N. de, Santos T. de O., Souza
Neto J. D. de, Riva-Souza E. M., Posse S. C. P., Souza M. N., Madella de Oliveira A. de F., Santos Júnior A. C., Santos J. O. (2024). Genetic diversity of common bean (*Phaseolus
vulgaris* L.) landraces based on morphological traits and
molecular markers. Plants.

[4] Cortinovis G., Vincenzi L., Anderson R., Marturano G., Marsh J. I., Bayer P. E. (2024). Adaptive
gene loss in
the common bean pan-genome during range expansion and domestication. Nat. Commun..

[5] de
Juan A., de Oliveira R. R. (2026). Hyperspectral image and chemometrics:
a step beyond classical spectroscopic PAT tools. Anal. Bioanal. Chem..

[6] Tafiire H., Wainaina I. N., Lugumira R., An N. T. H., Ogwok P., Grauwet T. (2024). A detailed
study on the cooking kinetics of fresh and
hard-to-cook common beans (*Phaseolus vulgaris* L.):
a case study on bean accessions of different market classes. J. Food Eng..

[7] de
Sousa M., Morales C. F. G., Mbanjo E. G. N., Egesi C., Oliveira E. J. de (2024). Near infrared spectroscopy for cooking time classification
of cassava genotypes. Front. Plant Sci..

[8] Rashvand M., Paterna G., Laveglia S., Zhang H., Shenfield A., Gioia T. (2025). Quality
detection of common bean flour using hyperspectral
imaging technology: potential of machine learning and deep learning. J. Food Compos. Anal..

[9] Zhu Y., Fan S., Zuo M., Zhang B., Zhu Q., Kong J. (2024). Discrimination
of new and aged seeds based on on-line near-infrared spectroscopy
technology combined with machine learning. Foods.

[10] Ram B. G., Oduor P., Igathinathane C., Howatt K., Sun X. (2024). A systematic
review of hyperspectral imaging in precision agriculture: Analysis
of its current state and future prospects. Comput.
Electron. Agric..

[11] Li S., Sun L., Jin X., Feng G., Zhang L., Bai H. (2025). Research on variety identification of common bean seeds
based on
hyperspectral and deep learning. Spectrochim.
Acta, Part A: Mol. Biomol. Spectrosc..

[12] Bitocchi E., Nanni L., Bellucci E., Rossi M., Giardini A., Zeuli P. S. (2012). Mesoamerican
origin of the common bean (*Phaseolus vulgaris* L.)
is revealed by sequence data. Proc. Natl. Acad.
Sci. U.S.A..

[13] Beć K. B., Grabska J., Huck C. W. (2025). Handheld NIR spectroscopy for real-time
on-site food quality and safety monitoring. Adv. Food Nutr. Res..

[14] Chorianopoulos N., Lytou A., Fengou L.-C., Kinoshita S., Dong P., Zhang Y. (2025). From lab
to market:
Real-time food safety monitoring via spectroscopy, blockchain and
artificial intelligence. Trends Food Sci. Technol..

[15] Jin C., Zhou L., Pu Y., Zhang C., Qi H., Zhao Y. (2025). Application of deep
learning for high-throughput phenotyping of seed:
A review. Artif. Intell. Rev..

[16] Ahmed M. T., Ahmed M. W., Kamruzzaman M. (2025). A systematic
review of explainable
artificial intelligence for spectroscopic agricultural quality assessment. Comput. Electron. Agric..

[17] Gorde P. M., Singha P., Singh S. K. (2025). Advanced
machine learning techniques
for hyacinth bean identification using infrared spectroscopy and computer
vision. Sustain. Food Technol..

[18] Bushuiev R., Bushuiev A., Samusevich R., Brungs C., Sivic J., Pluskal T. (2026). Self-supervised learning
of molecular representations
from millions of tandem mass spectra using DreaMS. Nat. Biotechnol..

[19] Özdoğan G., Lin X., Sun D.-W. (2021). Rapid and noninvasive sensory analyses of food products
by hyperspectral imaging: Recent application developments. Trends Food Sci. Technol..

[20] Yu G., Ma B., Li Y., Dong F. (2024). Quality detection of watermelons
and muskmelons using innovative nondestructive techniques: A comprehensive
review of novel trends and applications. Food
Control.

[21] Yang C., Guo Z., Fernandes Barbin D., Dai Z., Watson N., Povey M. (2025). Hyperspectral imaging and deep learning for quality
and safety inspection of fruits and vegetables: A review. J. Agric. Food Chem..

[22] Xiao Y., Zhou L., Zhao Y., Qi H., Pu Y., Zhang C. (2025). Deep learning-based regression of food quality attributes
using near-infrared
spectroscopy and hyperspectral imaging: A review. Food Chem..

[23] Gariso R., Coutinho J. P. L., Rato T. J., Reis M. S. (2025). A comparative analysis
of deep learning and chemometric approaches for spectral data modeling. Anal. Chim. Acta.

[24] Wong C. Y. S., Gilbert M. E., Pierce M. A., Parker T. A., Palkovic A., Gepts P., Magney T. S., Buckley T. N. (2023). Hyperspectral
remote
sensing for phenotyping the physiological drought response of common
and tepary bean. Plant Phenomics.

[25] Hassanzadeh A., Murphy S. P., Pethybridge S. J., van Aardt J. (2020). Growth stage
classification and harvest scheduling of snap bean using hyperspectral
sensing: A greenhouse study. Remote Sens..

[26] Kior A., Sukhov V., Sukhova E. (2021). Application
of reflectance indices
for remote sensing of plants and revealing actions of stressors. Photonics.

[27] Wang D., Cao W., Zhang F., Li Z., Xu S., Wu X. (2022). A review of
deep learning in multiscale agricultural sensing. Remote Sens..

[28] Zhang G., Abdulla W. (2023). Explainable AI-driven
wavelength selection for hyperspectral
imaging of honey products. Food Chem. Adv..

[29] Li K.-Y., Sampaio de Lima R., Burnside N. G., Vahtmäe E., Kutser T., Sepp K., Cabral Pinheiro V. H., Yang M.-D., Vain A., Sepp K. (2022). Toward automated
machine
learning-based hyperspectral image analysis in crop yield and biomass
estimation. Remote Sens..

[30] Contreras J., Bocklitz T. (2025). Explainable artificial
intelligence for spectroscopy
data: A review. Pflugers Arch..

[31] Braga P., Crusiol L. G. T., Nanni M. R., Caranhato A. L. H., Fuhrmann M. B., Nepomuceno A. L. (2021). Vegetation indices and
NIR-SWIR spectral bands as a phenotyping tool for water status determination
in soybean. Precis. Agric..

[32] Dhanya V. G., Subeesh A., Susmita C., Amaresh, Saji S. J., Dilsha C. (2024). High-throughput phenotyping using hyperspectral imaging for seed
quality assurance coupled with machine learning methods: Principles
and way forward. Plant Physiol. Rep..

[33] Yoosefzadeh-Najafabadi M., Tulpan D., Eskandari M. (2021). Using hybrid artificial intelligence
and evolutionary optimization algorithms for estimating soybean yield
and fresh biomass using hyperspectral vegetation indices. Remote Sens..

[34] Wang L., Gao R., Li C., Wang J., Liu Y., Hu J., Li B., Qiao H., Feng H., Yue J. (2023). Mapping soybean maturity
and biochemical traits using UAV-based hyperspectral images. Remote Sens..

[35] Phaneendra
Kumar B. L. N., Vaddi R., Manoharan P., Agilandeeswari L., Sangeetha V. (2024). A new band selection framework for
hyperspectral remote sensing image classification. Sci. Rep..

[36] Choi J., Nam G., Choi J., Jung Y. (2025). A perspective on foundation models
in chemistry. JACS Au.

[37] Alberts M., Laino T., Vaucher A. C. (2024). Leveraging
infrared spectroscopy
for automated structure elucidation. Commun.
Chem..

[38] Flanagan A. R., Dalal D., Glavin F. G. (2025). Exploring
generative artificial intelligence
and data augmentation techniques for spectroscopy analysis. Chem. Rev..

[39] Tsoumas I., Sitokonstantinou V., Giannarakis G., Lampiri E., Athanassiou C., Camps-Valls G. (2025). Leveraging causality and explainability in
digital agriculture. Environ. Data Sci..

[40] Farooq M. A., Gao S., Hassan M. A., Huang Z., Rasheed A., Hearne S. (2024). Artificial
intelligence in plant breeding. Trends Genet..

[41] Kiratiratanapruk K., Temniranrat P., Sinthupinyo W., Prempree P., Chaitavon K., Porntheeraphat S. (2020). Development of paddy rice seed classification
process using machine learning techniques for automatic grading machine. J. Sensors.

[42] Jiao L., Wang Y., Liu X., Li L., Liu F., Ma W. (2024). Causal inference meets
deep learning: A comprehensive
survey. Research.

[43] Wu S., Liu Y., Fan X., Shen Y., Qu H. (2025). Trends and new process
analytical technologies in pharmaceutical manufacturing. Int. J. Pharm..

[44] Pedregosa F., Varoquaux G., Gramfort A., Michel V., Thirion B., Grisel O. (2011). Scikit-learn: machine
learning in Python. J. Mach. Learn. Res..

[45] Jeong S. W., Lyu J. I., Jeong H., Baek J., Moon J.-K., Lee C. (2024). SUnSeT:
spectral unmixing of hyperspectral images for
phenotyping soybean seed traits. Plant Cell
Rep..

[46] Ahmad M., Distefano S., Khan A. M., Mazzara M., Li C., Li H. (2025). A comprehensive survey for hyperspectral image classification:
The evolution from conventional to transformers and Mamba models. Neurocomputing.

[47] Guardado
Yordi E., Koelig R., Matos M. J., Pérez
Martínez A., Caballero Y., Santana L., Pérez
Quintana M., Molina E., Uriarte E. (2019). Artificial intelligence
applied to flavonoid data in food matrices. Foods.

[48] Tan Y., Gu J., Lu L., Zhang L., Huang J., Pan L., Lv Y., Wang Y., Chen Y. (2025). Hyperspectral band selection for
crop identification and mapping of agriculture. Remote Sens..

[49] Shorten P.
R., Leath S. R., Schmidt J., Ghamkhar K. (2019). Predicting the quality
of ryegrass using hyperspectral imaging. Plant
Methods.

[50] Olmos V., Marro M., Loza-Alvarez P., Raldúa D., Prats E., Padrós F. (2018). Combining hyperspectral
imaging and chemometrics to assess and interpret the effects of environmental
stressors on zebrafish eye images at tissue level. J. Biophotonics.

