# Supplementary material for: Visible-Near-Infrared Hyperspectral Imaging Enables Nondestructive Identification of Bean Accessions via 1D Spectral Reflectance Analysis
Source: ACS Omega. 2026 May 7;11(19):28415–31. doi: 10.1021/acsomega.6c00225 (PMC13191541; doi:10.1021/acsomega.6c00225)
Supplement: Supplementary file 1 [file ao6c00225_si_001.pdf]

Supplementary files

VNIR hyperspectral imaging enables non-destructive identification of bean accessions via 1D spectral reflectance analysis

Renan Falcioni[1,2*], Nicole Ghinzelli Vedana[1], Caio Almeida de Oliveira[1], João Vitor Ferreira Gonçalves[1], José Alexandre M. Demattê[3] and Marcos Rafael Nanni[1]

[1] Graduate Program in Agronomy, State University of Maringá, Av. Colombo, 5790, Maringá 87020−900, Paraná, Brazil

[2] Department of Biology, State University of Maringá, Av. Colombo, 5790, Maringá 87020−900, Paraná, Brazil

[3] Department of Soil Science, Luiz de Queiroz College of Agriculture, University of São Paulo, Av. Pádua Dias, 11, Piracicaba 13418−260, São Paulo, Brazil

[*]Correspondence:
Renan Falcioni; renanfalcioni@gmail.com or rfalcioni2@uem.br

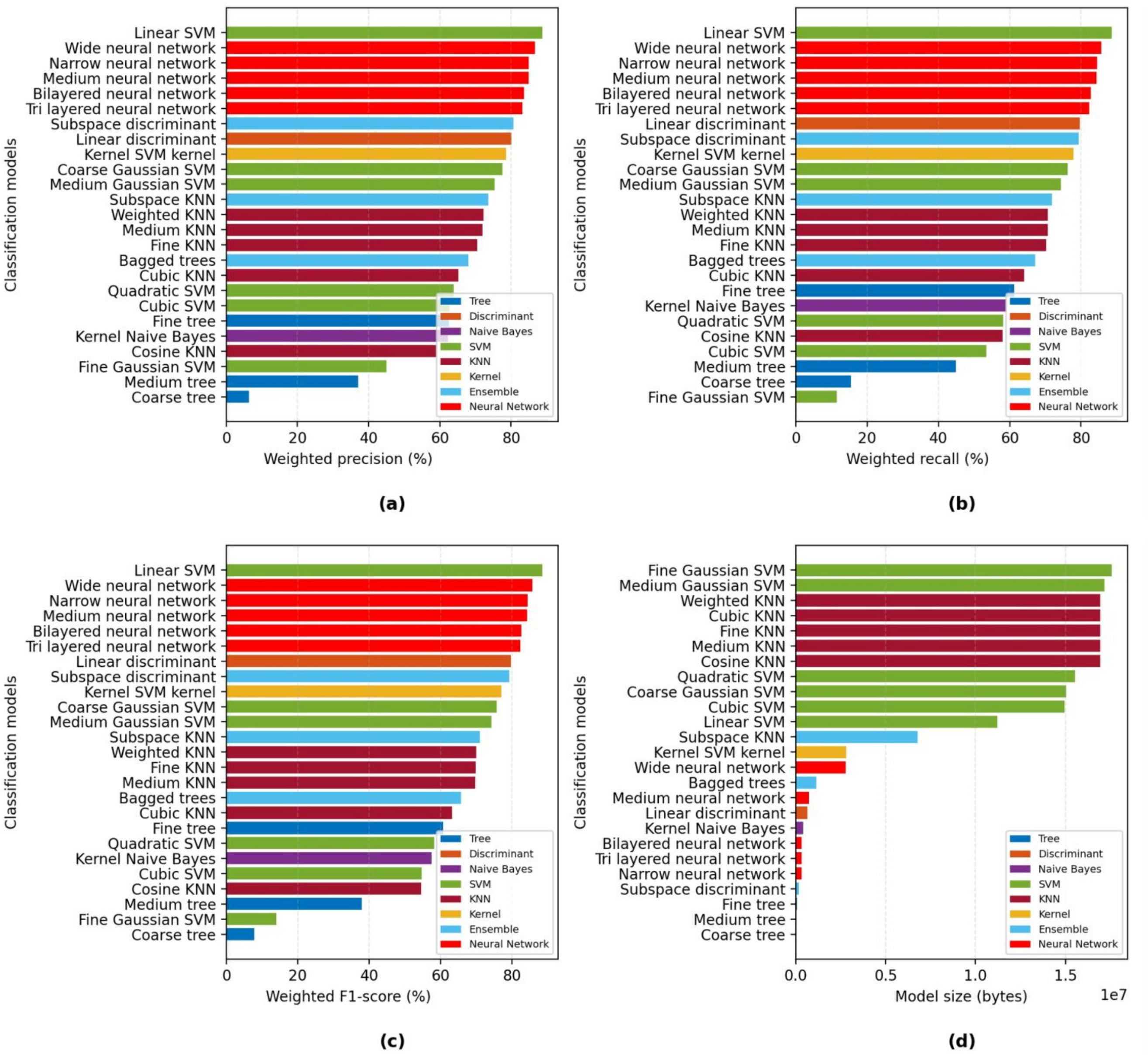

Figure S1. Performance on the independent 20% test split (80/20 hold-outs) of the 25 classification models used to discriminate the 32 grain-legume accessions from the spectral feature set. Weighted precision (a), weighted recall (b) and the weighted F1-score (c) are shown alongside the corresponding stored model size (d), where weighting accounts for class prevalence and therefore reflects performance under potential class imbalance. Abbreviations: KNN, k-nearest neighbours; SVM, support vector machine.

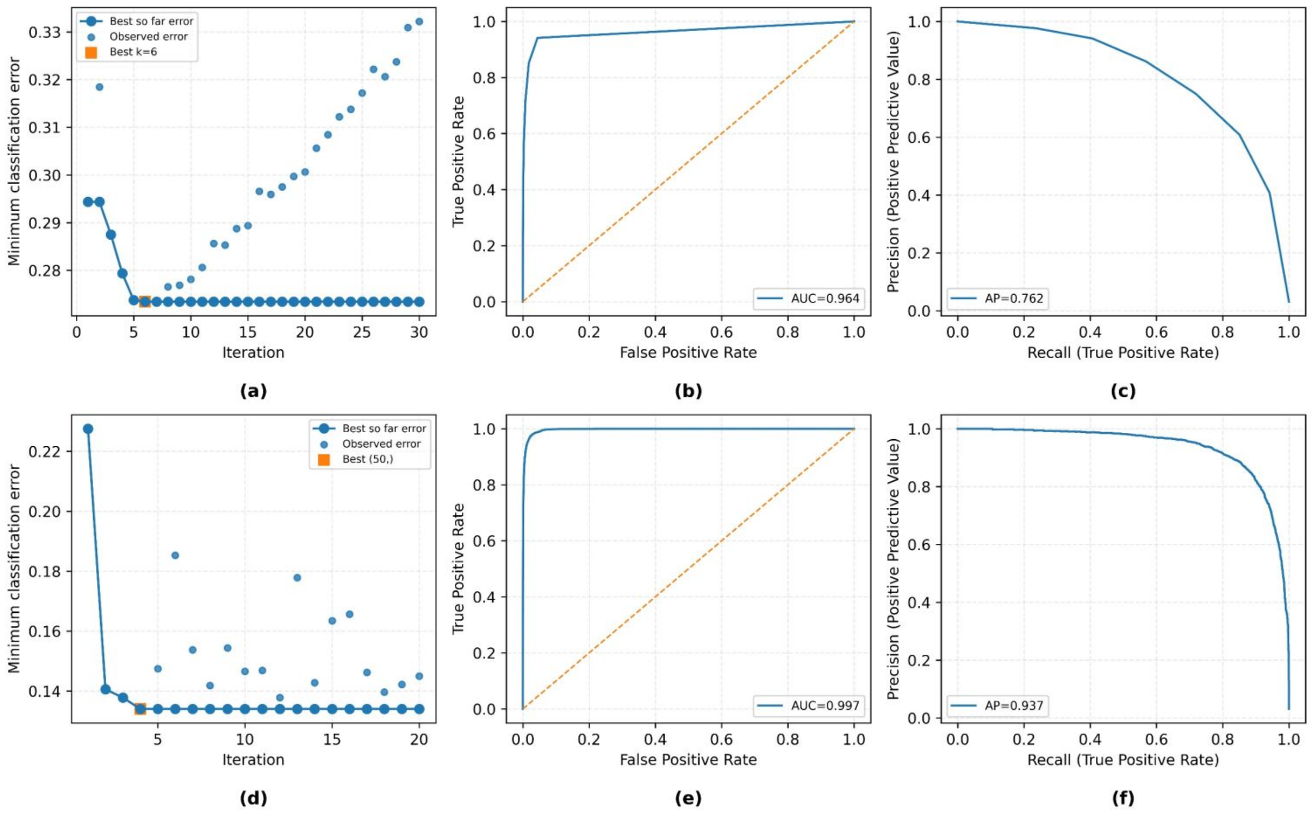

Figure S2. Hyperparameter optimisation traces and validation operating characteristics for the two optimised classifiers. (a–c) correspond to the KNN optimisation, showing the evolution of the minimum classification error over optimisation iterations (a), the resulting validation ROC curve with its area under the curve (AUC) (b), and the validation precision–recall curve summarised by average precision (AP) (c). Panels (d–f) provide the analogous outputs for the optimised neural-network classifier, including the minimum classification error trajectory (d), ROC/AUC (e) and precision–recall/AP (f).

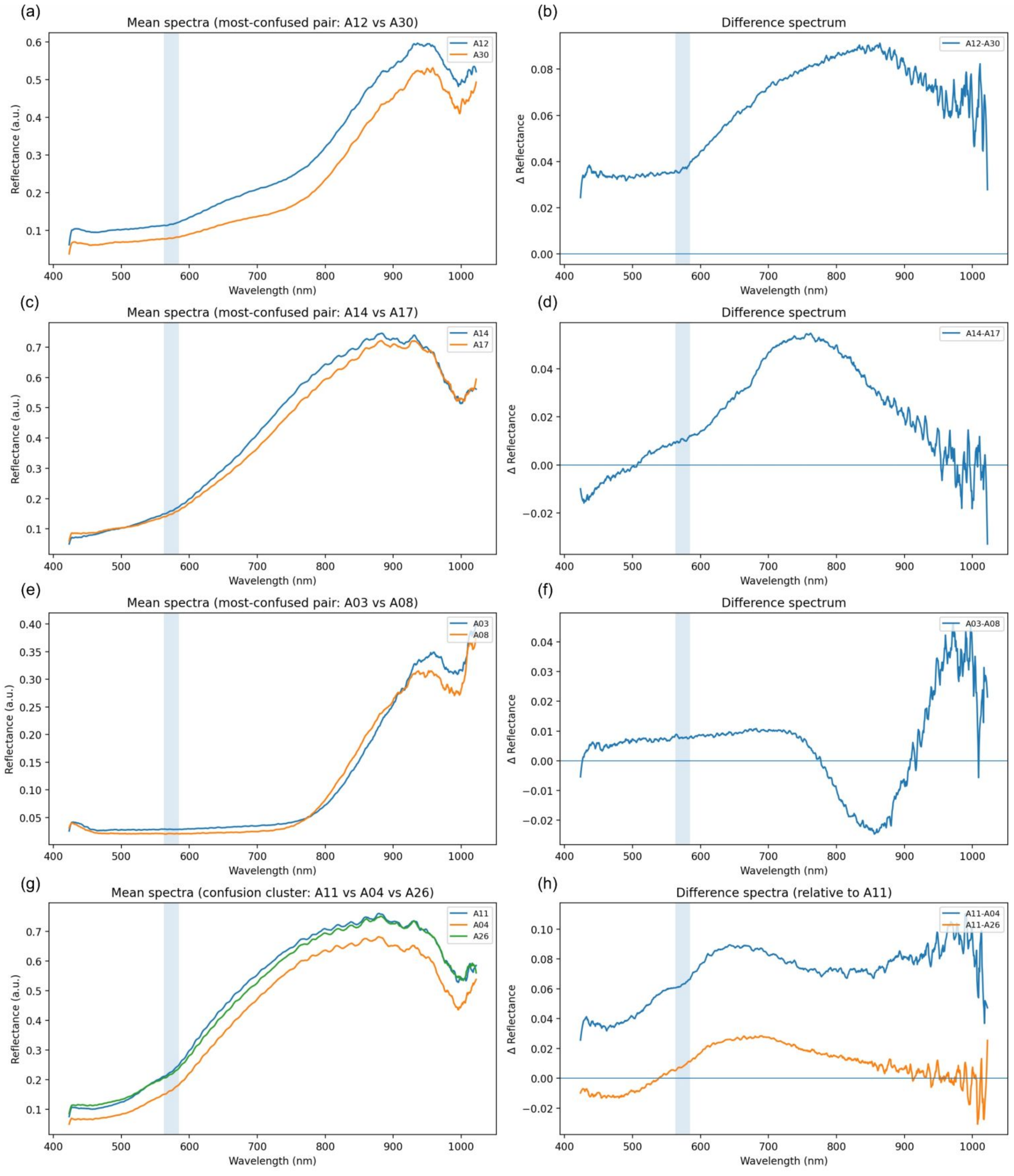

Figure S3. Mean pre-processed reflectance spectra (left) and difference spectra (right; $\Delta = \mathrm{A} - \mathrm{B}$) for the accession pairs most frequently confused by the linear SVM classifier. The shaded band indicates the green window (562.85–584.65 nm) identified as a dominant discriminative interval by wavelength-wise association mapping and ReliefF.

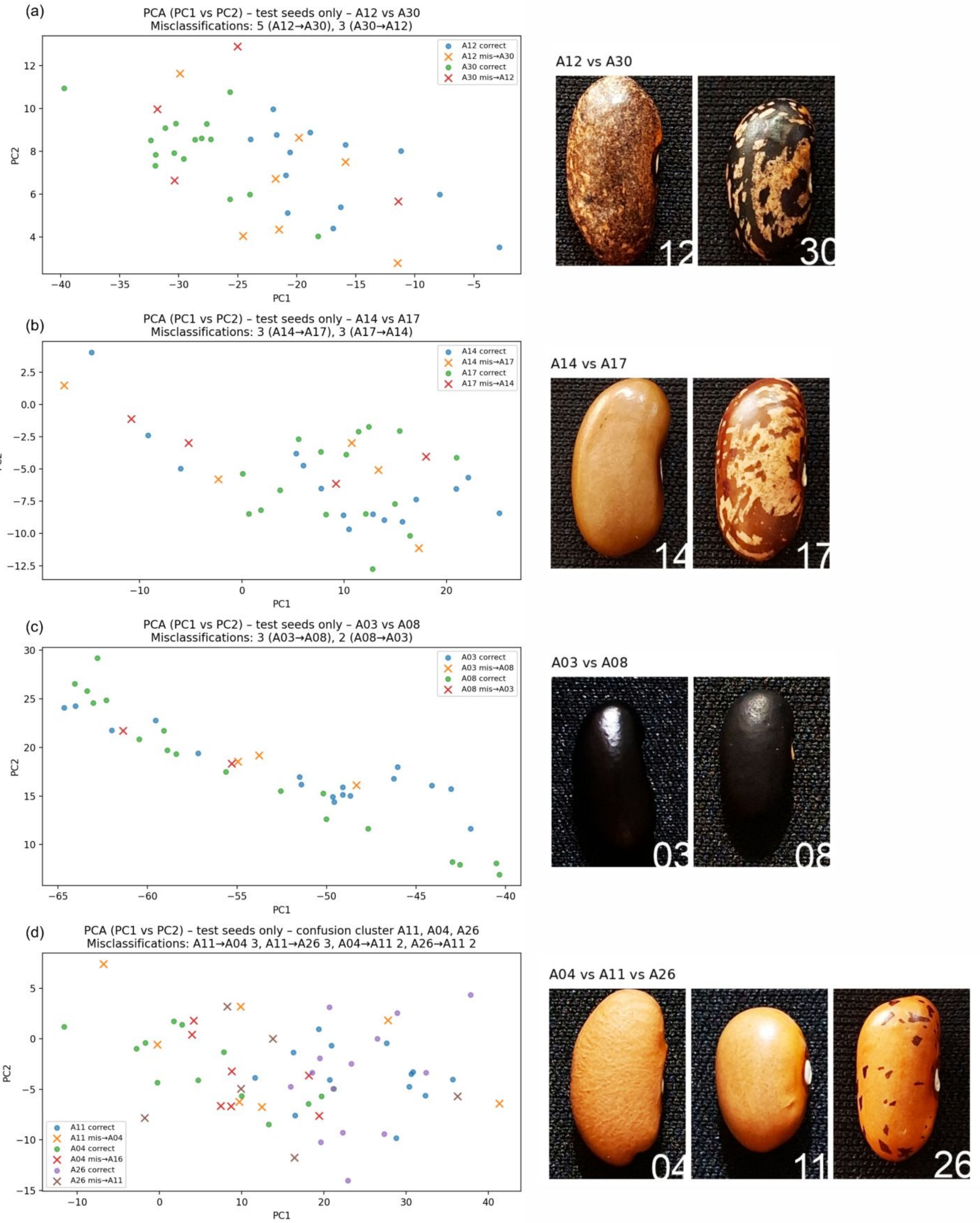

Figure S4. PCA score plots (PC1 vs PC2) for test-set seeds from the accession pairs most frequently confused by the linear SVM classifier. The circles denote correctly classified seeds, and the crosses denote misclassified seeds (the misclassification direction is indicated in the legend). Representative seed images are shown for each confused pair/cluster.

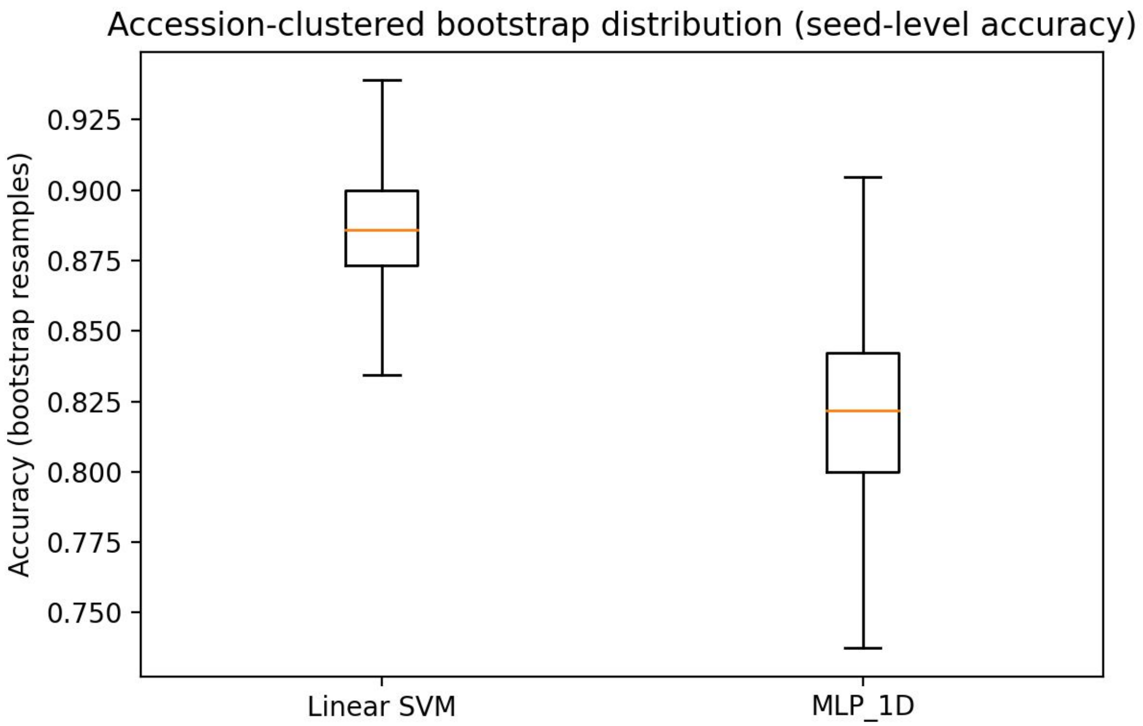

Figure S5. Accession-clustered bootstrap distributions of seed-level accuracy for the top classical (linear SVM) and top deep (MLP_1D) models. Bootstrap resampling was performed at the accession (cluster) level to account for intra-accession correlations among seeds.

Table S1. A complete list of thirty common bean landraces (*Phaseolus vulgaris* L.) and two non-Phaseolus outgroup legumes (*Vigna angularis* (Willd.) Ohwi & H. Ohashi and *Cajanus cajan* (L.) Huth), with accession codes and seed phenotype descriptions used in this study. Descriptions reflect recorded cultivar names or batch-level visual phenotypes where no standard description was available.

Abbreviation[1]	Accession name[2]	Characteristics of beans[3]
A01	Feijão Amendoim Roxo	Purple/violet seed coat; elongated ‘peanut-type’ seeds; uniform pattern.
A02	Feijão Carioca	Cream/beige seed coat with brown stripes (carioca type); kidney-shaped seeds; medium size.
A03	Feijão Preto Gigante	Black seed coat; large seeds; uniform pattern.
A04	Feijão Branco Manteiga	White/pale cream seed coat (butter type); large seeds; uniform pattern.
A05	Feijão Rosinha	Pink seed coat; medium-sized seeds; uniform pattern.
A06	Feijão Branco Gigante	White seed coat; very large seeds; uniform pattern.
A07	Feijão Canário	Canary-yellow seed coat; medium-sized seeds; uniform pattern.
A08	Feijão Preto Mocotó	Black seed coat; medium-sized seeds; uniform pattern.
A09	Feijão Verde	Green seed coat (olive to light green); medium-sized seeds; uniform pattern.
A10	Feijão Vermelho Bolinha	Red seed coat; small, rounded (‘little ball’) seeds; uniform pattern.
A11	Feijão Branco Bolinha	White seed coat; small, rounded seeds; uniform pattern.
A12	Feijão Mouro Gigante	Dark seed coat (mouro: dark brown to black); large seeds; uniform to slightly mottled pattern.
A13	Feijão Roxinho	Purplish-red seed coat; medium-sized seeds; uniform pattern.
A14	Feijão Creme	Cream/light beige seed coat; medium-sized seeds; uniform pattern.
A15	Feijão Vermelho Gigante	Red seed coat; large seeds; uniform pattern.
A16	Feijão Jalo	Golden-yellow seed coat (jalo type); large seeds; uniform pattern.
A17	Feijão Amendoim Bege	Beige seed coat; elongated ‘peanut-type’ seeds; uniform pattern.
A18	Feijão Boreal Rosa	Pink seed coat; medium-sized seeds; uniform pattern.
A19	Feijão Chocolate	Chocolate-brown seed coat; medium-sized seeds; uniform pattern.
A20	Feijão Amendoim Rosa	Pink seed coat; elongated ‘peanut-type’ seeds; uniform pattern.

A21	Feijão Olho de Pombo	Cream seed coat with a dark hilum (‘eye’); bicolour ‘pigeon-eye’ pattern; small to medium seeds.
A22	Feijão Bico de Ouro	Yellow/golden seed coat (‘bico-de-ouro’ type); medium-sized seeds; uniform pattern.
A23	Feijão Amendoim	Beige to light-brown seed coat; elongated ‘peanut-type’ seeds; uniform pattern.
A24	Feijão Ovo de Tito-Tico	Light-coloured seed coat with contrasting speckles/blotches (‘tico-tico egg’ type); small, oval seeds.
A25	Feijão Amendoim Verde	Green seed coat; elongated ‘peanut-type’ seeds; uniform pattern.
A26	Feijão Rajado	Light seed coat with darker streaks/blotches (striped/mottled); medium-sized seeds; variegated pattern.
A27	Feijão Azaki	Azuki (*Vigna angularis* (Willd.) Ohwi & H. Ohashi): typically uniform red seed coat; small seeds.
A28	Feijão Guandu	Pigeon pea (*Cajanus cajan* (L.) Huth): rounded seeds; cream to brown seed coat; typically uniform pattern.
A29	Feijão Mouro	Dark seed coat (mouro); small to medium seeds; uniform pattern.
A30	Feijão Mancá	Local landrace (‘Mancá’): based on the observed seed colour/pattern of the batch (no standard description available).
A31	Feijão Vermelho	Red seed coat; medium-sized seeds; uniform pattern.
A32	Feijão Rapa Cuiua	Rapa Cuia/Rapa Cuiua (*Phaseolus vulgaris* L.): traditional landrace; associated with dark/black beans; dark seeds.

[1]Abbreviation (ID): identification code used to trace accessions through spectral extraction and modelling. [2]Accession name: common/popular name of each accession. [3]Characteristics of beans: morphological description of the analysed accessions on the basis of the imaged batches.

Table S2. Classical model performance on the primary stratified 80/20 hold-out evaluation (with accession-clustered uncertainty). Each accession contributed 80 seeds to the training set and 20 seeds to the independent test set ($n\ test$ = 640; 32 accessions × 20 seeds). SeedAcc and MacroF1 were computed at the seed level. The 95% confidence intervals for Seed Acc and MacroF1 were estimated via accession-clustered bootstrap resampling (clusters = accessions; G = 32) to account for intra-accession correlations among seeds. LineAcc reports accession-level identification accuracy obtained by majority vote across the 20 test seeds within each accession. For LineAcc, k is the number of correctly identified accessions out of n = 32, and confidence intervals are exact binomial 95% CIs. All values are reported as percentages.

Model	Seed Acc	Seed Acc CI low	Seed Acc CI high	MacroF1	MacroF1 CI low	MacroF1 CI high	Line Acc	Line Acc CI low	Line Acc CI high	k	n
Fine tree	61.250	53.438	69.063	60.817	55.369	66.170	93.750	79.193	99.234	30	32
Medium tree	44.844	31.563	58.281	38.018	29.080	46.863	56.250	37.663	73.636	18	32
Coarse tree	15.469	3.125	27.969	7.874	1.857	12.769	15.625	5.275	32.788	5	32
Linear discriminant	79.688	73.281	85.625	79.756	75.064	84.596	100.000	89.112	100.000	32	32
Kernel Naive Bayes	59.062	50.000	67.969	57.499	51.547	63.443	81.250	63.561	92.792	26	32
Linear SVM	88.594	84.531	92.344	88.517	85.884	91.197	100.000	89.112	100.000	32	32
Quadratic SVM	58.125	47.969	67.812	58.214	51.113	65.407	75.000	56.595	88.538	24	32
Cubic SVM	53.438	42.344	64.375	54.761	46.507	63.061	68.750	49.992	83.882	22	32
Fine Gaussian SVM	11.562	6.562	18.594	14.041	10.429	17.531	3.125	0.079	16.217	1	32
Medium Gaussian SVM	74.375	68.437	80.156	74.233	70.223	78.335	96.875	83.783	99.921	31	32
Coarse Gaussian SVM	76.250	69.844	82.188	75.686	71.873	79.645	96.875	83.783	99.921	31	32
Fine KNN	70.156	62.969	76.875	69.839	64.960	74.801	93.750	79.193	99.234	30	32
Medium KNN	70.625	63.125	77.656	69.772	65.194	74.402	90.625	74.977	98.023	29	32
Cosine KNN	57.969	48.125	67.344	54.644	49.685	59.838	75.000	56.595	88.538	24	32
Cubic KNN	64.062	56.563	71.406	63.288	58.380	68.163	93.750	79.193	99.234	30	32
Weighted KNN	70.625	63.594	77.344	70.011	65.725	74.240	96.875	83.783	99.921	31	32
Bagged trees	67.188	58.906	75.000	65.751	60.944	70.620	93.750	79.193	99.234	30	32
Subspace discriminant	79.375	73.750	84.535	79.254	75.723	82.809	96.875	83.783	99.921	31	32
Subspace KNN	71.875	64.687	78.750	70.987	66.833	75.133	93.750	79.193	99.234	30	32

Narrow neural network	84.531	79.062	89.375	84.417	80.945	88.087	100.000	89.112	100.000	32	32
Medium neural network	84.375	79.688	88.750	84.290	81.305	87.391	96.875	83.783	99.921	31	32
Wide neural network	85.625	80.781	90.156	85.745	82.438	89.183	100.000	89.112	100.000	32	32
Bilayered neural network	82.656	77.812	87.188	82.599	79.473	85.801	100.000	89.112	100.000	32	32
Tri layered neural network	82.344	77.500	86.719	82.353	79.128	85.707	100.000	89.112	100.000	32	32
Kernel SVM	77.812	71.094	83.750	77.121	73.309	81.139	93.750	79.193	99.234	30	32

Table S3. Classical model performance under stratified K-fold cross-validation (with accession-clustered uncertainty). Models were evaluated via stratified K-fold cross-validation (K = 5), with splits stratified by accession so that all 32 accessions were represented in every fold and each test fold comprised 20 seeds per accession. Seed Acc and MacroF1 summarise the fold-aggregated seed-level performance. 95% confidence intervals were estimated via accession-clustered bootstrap resampling (clusters = accessions; G = 32), ensuring uncertainty quantification that respects within-accession dependence. Line Acc summarises accession-level performance via majority vote within each accession from out-of-fold predictions; *k* and *n* and the exact binomial 95% CI are defined as in Table S2. All values are reported as percentages.

Model	Seed Acc	Seed Acc CI low	Seed Acc CI high	MacroF1	MacroF1 CI low	MacroF1 CI high	Line Acc	Line Acc CI low	Line Acc CI high	k	n
Fine tree	63.969	58.563	69.594	64.067	58.713	69.665	100.000	89.112	100.000	32	32
Medium tree	46.281	36.281	56.282	43.461	34.321	52.725	62.500	43.692	78.900	20	32
Coarse tree	15.250	5.469	27.000	9.408	2.786	17.711	15.625	5.275	32.788	5	32
Linear discriminant	79.750	74.031	85.251	79.744	74.090	85.048	100.000	89.112	100.000	32	32
Kernel Naive Bayes	58.906	50.375	67.281	57.234	49.876	64.568	87.500	71.005	96.487	28	32
Linear SVM	85.219	80.625	89.375	85.125	80.727	89.030	100.000	89.112	100.000	32	32
Quadratic SVM	74.125	68.000	79.938	73.686	68.509	78.614	96.875	83.783	99.921	31	32
Cubic SVM	80.188	73.875	85.719	79.668	74.128	84.417	96.875	83.783	99.921	31	32
Fine Gaussian SVM	78.688	73.093	83.907	78.508	73.412	83.173	96.875	83.783	99.921	31	32
Medium Gaussian SVM	68.031	60.094	75.313	66.611	59.773	72.912	90.625	74.977	98.023	29	32
Coarse Gaussian SVM	49.906	39.813	59.969	46.441	37.895	54.745	65.625	46.807	81.428	21	32
Fine KNN	70.531	64.781	76.219	70.556	64.900	76.167	96.875	83.783	99.921	31	32
Medium KNN	70.594	64.124	76.906	69.986	64.520	75.311	96.875	83.783	99.921	31	32
Cosine KNN	73.344	66.000	80.094	72.857	67.044	78.382	90.625	74.977	98.023	29	32
Weighted KNN	72.500	66.500	78.375	72.072	67.014	76.961	96.875	83.783	99.921	31	32
Bagged trees	69.438	63.406	75.094	68.700	63.849	73.568	96.875	83.783	99.921	31	32
Subspace discriminant	80.250	74.500	85.531	79.843	75.162	84.202	100.000	89.112	100.000	32	32
Subspace KNN	72.125	65.344	78.563	71.359	65.688	76.680	93.750	79.193	99.234	30	32
Narrow neural network	63.375	56.094	70.438	62.782	56.338	69.320	96.875	83.783	99.921	31	32

Medium neural network	84.969	80.844	88.844	84.887	81.032	88.537	100.000	89.112	100.000	32	32
Wide neural network	86.813	83.000	90.220	86.781	83.312	89.981	100.000	89.112	100.000	32	32
Bilayered neural network	56.031	50.155	61.875	55.675	50.337	61.263	96.875	83.783	99.921	31	32
Tri layered neural network	80.938	76.656	85.156	80.814	76.816	84.796	100.000	89.112	100.000	32	32
Kernel SVM	56.313	45.655	67.031	52.243	44.761	59.418	78.125	60.027	90.723	25	32

Note: Cubic KNN was not included in the cross-validation experiment and is therefore reported only in the hold-out benchmark (Figure 5 and Table S2).

Table S4. Comparison of spectral band-selection strategies for seed classification, including the number of selected wavelength bands and the corresponding classification performance measured by accuracy (%) and macro-F1 (%).

Selection	Bands	Seed Acc (%)	Macro F1 (%)
Full spectrum (all bands)	825	88.594	88.517
ReliefF top-16 wavelengths	16	31.875	28.201
Green window 562.85–584.65 nm	31	34.375	31.196
Uniform sampling every 30 nm	20	83.125	82.934
Uniform sampling every 20 nm	30	84.844	84.772
Uniform sampling every 10 nm	60	87.500	87.394
Uniform sampling every 5 nm	120	89.063	89.001
